# LiFePO_4_ Battery Material for the Production of Lithium from Brines: Effect of Brine Composition and Benefits of Dilution

**DOI:** 10.1002/cssc.202102182

**Published:** 2021-11-24

**Authors:** Sara Pérez‐Rodríguez, Samuel D. S. Fitch, Philip N. Bartlett, Nuria Garcia‐Araez

**Affiliations:** ^1^ Department of Chemistry University of Southampton University Road Southampton SO171BJ United Kingdom

**Keywords:** aqueous lithium-ion batteries, brine, electrochemistry, lithium, lithium sequestration

## Abstract

Lithium battery materials can be advantageously used for the selective sequestration of lithium ions from natural resources, which contain other cations in high excess. However, for practical applications, this new approach for lithium production requires the battery host materials to be stable over many cycles while retaining the high lithium selectivity. Here, a nearly symmetrical cell design was employed to show that LiFePO_4_ shows good capacity retention with cycling in artificial lithium brines representative of brines from Chile, Bolivia and Argentina. A quantitative correlation was identified between brine viscosity and capacity degradation, and for the first time it was demonstrated that the dilution of viscous brines with water significantly enhanced capacity retention and rate capability. The electrochemical and X‐ray diffraction characterisation of the cycled electrodes also showed that the high lithium selectivity was preserved with cycling. Raman spectra of the cycled electrodes showed no signs of degradation of the carbon coating of LiFePO_4_, while scanning electron microscopy images showed signs of particle cracking, thus pointing towards interfacial reactions as the cause of capacity degradation.

## Graphical abstract

**Brine is fine**: The electrochemical sequestration of lithium from brines representative of the largest lithium resources in South America is explored, using a battery host material (LiFePO_4_) as a sustainable approach of lithium production. The brine viscosity is found to critically affect the cycling stability and rate capability, and, surprisingly, significant improvements are achieved by simple brine dilution. Post‐mortem characterisation suggests that interfacial side‐reactions and particle cracking limit the long‐term stability.

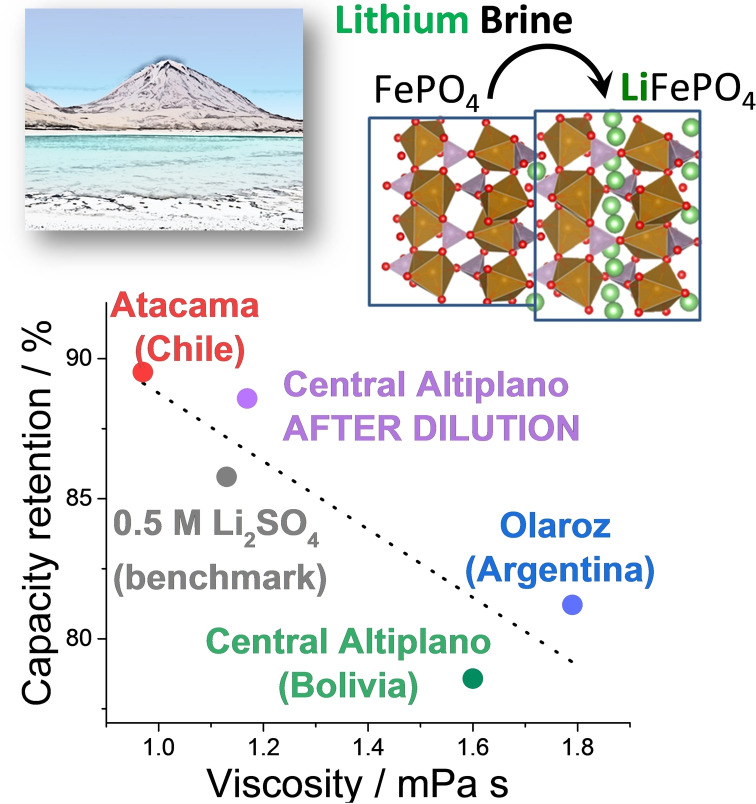

## Introduction

The demand for lithium production has increased dramatically in recent years, due to the expansion of lithium battery applications in electric vehicles, portable devices and large‐scale energy storage, and an even greater demand for lithium is expected in coming years.[^1^, ^2^, ^3^, ^4^, ^5^] In order to satisfy this ever‐increasing demand, the development of alternative methods for lithium production is urgently needed in order to replace or complement the current lithium production technologies and thus enable a sustainable expansion in the lithium production without significantly increasing the cost or producing a detrimental environmental impact.[^6^, ^7^, ^8^, ^9^]

Most world lithium resources are in the form of brines, which are concentrated saline solutions containing lithium cations in the presence of a vast excess of other cations (called “co‐cations”) such as Na^+^, K^+^ and Mg^2+^.[^6^, ^8^] Current methods of lithium production from brines are based on a solar evaporation/precipitation technology (lima‐soda evaporation), which is time‐consuming, involves the use of a considerable amount of water and generates a significant volume of waste.^[6]^ In addition, unfortunately, the lima‐soda evaporation can only be applied to a narrow range of brine compositions.^[6]^

**Figure 1:**
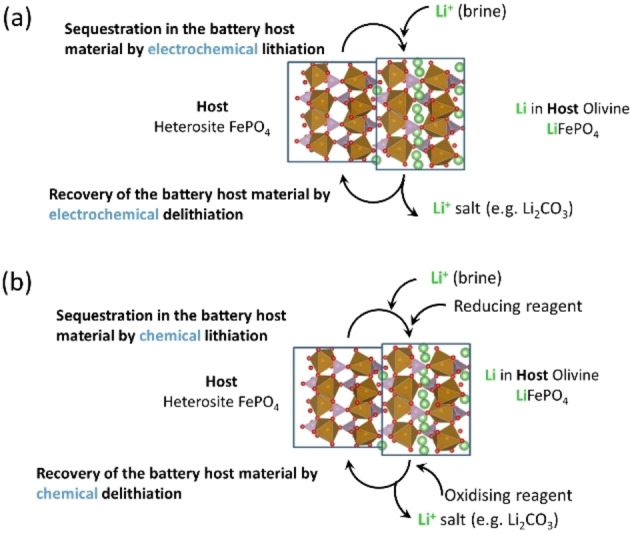
Illustration of the method of lithium sequestration with battery materials by employing (a) electricity or (b) redox agents to drive the reactions of lithium insertion and extraction from the battery host material, which in this case is FePO_4_.

Therefore, the development of alternative methods of lithium production has been intensively researched in recent years. Several methods of lithium production have been explored such as solvent extraction using novel organic systems, ion‐sieve adsorption or membrane technology.[^6^, ^7^, ^8^, ^10^, ^11^] A particularly promising approach is the use of lithium battery materials, which results in an unprecedented selectivity towards lithium and, hence, enables the use of brines with very different compositions.[^12^, ^13^] In this approach, lithium ions in the brine are sequestrated by a host battery material, and subsequently, they are released in a clean, recovery solution, from which they can be precipitated producing a pure lithium salt, and in this last step the initial host battery material is also recovered and, thus, it can be used many times, making the whole process fully sustainable (see Figure 1). The sequestration and release of lithium in the battery material can be driven by electricity (Figure 1a) or redox agents (Figure 1b). The latter approach can produce faster reaction kinetics, but the cost and environmental compatibility of redox agents are critical factors that deserve further investigation.[^13^, ^14^] On the other hand, when the reactions are induced by electricity, the use of additional chemical species is avoided, thus producing cleaner reactions and minimising the formation of chemical waste.[^15^, ^16^, ^17^]

Kanoh et al.[^15^, ^16^] first demonstrated the selective electrochemical sequestration of lithium ions from aqueous solutions, containing different cations, into a battery material, and later work by La Mantia and co‐workers^[17]^ demonstrated the promise and viability of using a battery‐like electrochemical cell as a new and sustainable method of lithium production from brines. Since then, significant work has been dedicated to the investigation of the electrode materials to employ in the cell. The battery‐like electrochemical cell consists of a lithium‐selective battery electrode (e. g., LiFePO_4_,[^17^, ^18^, ^19^] λ‐MnO_2_[^20^, ^21^, ^22^, ^23^, ^24^, ^25^]) combined with a chloride capturing (e. g., Ag,[^17^, ^18^, ^20^, ^21^, ^22^, ^26^, ^27^] polyaniline,^[23]^ polypyrrole^[24]^) or a lithium‐exclusion (e. g., nickel hexacyanoferrate,[^25^, ^28^] ferrocyanide^[19]^) counter‐electrode. The selective extraction of lithium from brines is achieved because the lithium battery electrode undergoes a reduction reaction that induces the insertion of only lithium ions in the electrode, whereas the electrochemical reaction at the counter electrode does not involve lithium ions.

Although this approach is highly promising, the long‐term stability of the electrodes, under the operation conditions in the natural brines, and the preservation of the high lithium selectivity of the lithium battery electrode over many cycles, are critical requirements for the commercial viability of this new technology. Unfortunately, these aspects have received little attention. In addition, there is a lack of consistency in the experimental conditions employed in different articles, which hampers an accurate comparison of the results.[^12^, ^13^] A variety of solution compositions have been employed, with different concentrations of lithium and other cations, and different counter‐electrode materials and cell designs have been employed. All these factors critically affect the cycling stability and selectivity, as illustrated Tables S1 and S2.

In this work, we performed a systematic study of the cycling stability and lithium selectivity of LiFePO_4_ electrodes for the sequestration of lithium from solutions mimicking the composition of natural lithium brines. Contrary to previous studies in lithium brines,^[25]^ we have found that LiFePO_4_ exhibits good cycling stability under the brine operation conditions. We performed the experiments in three artificial brine compositions that represent typical compositions of the lithium reserves in Atacama (Chile),[^29^, ^30^] Olaroz (Argentina)[^24^, ^31^, ^32^] and Central Altiplano (Bolivia).^[33]^ Additional experiments were also done in Li_2_SO_4_ solutions, which serve as benchmark solutions containing only lithium and no other cations. Li_2_SO_4_ solutions have been used before in aqueous lithium‐ion batteries showing good cycling stability (see Table S1).

The comparison of behaviour of LiFePO_4_ in different brines revealed, for the first time, that the brine viscosity is a key factor affecting the process of lithium sequestration. In addition, we also demonstrate a new approach, using brine dilution, to enhance the lithium sequestration process, thus achieving longer cycling stability and improved rate capability.

The studies here reported were performed in a new electrochemical cell design with both electrodes made of the same battery material (LiFePO_4_, in different states of lithiation), so that any degradation with cycling could be directly ascribed to the degradation of this particular battery material. For the lithium production application, the electrochemical cell needs to incorporate a different material as counter electrode, as described above, so that the reactions in the cell produce the selective extraction of only lithium ions from the brine. However, under these conditions, the degradation of the cell performance with cycling can be due to the degradation of the counter or working electrodes or cross‐talk effects. Such ambiguity in the interpretation of results is overcome here by using a nearly symmetrical cell design, and characterisation techniques [X‐ray diffraction (XRD), Raman spectroscopy, scanning electron microscopy (SEM)] are applied to elucidate the mechanism of degradation.

## Results and Discussion

### Validation of the nearly symmetrical cell design

The new cell design developed here contains a LiFePO_4_ composite electrode acting as the working electrode and a partially‐delithiated Li_0.25_FePO_4_ composite electrode acting as both the counter electrode and reference electrode. The reactions induced with cycling are the following [Eqs. (1) and 2]:

(1) $$\mathrm{Working}\;\mathrm{electrode}:\;\mathrm{LiFePO}{}_{4}\vec{\leftarrow }\mathrm{FePO}{}_{4}+\mathrm{Li}{}^{+}+e{}^{-}$$

(2) $$\mathrm{Counter}\;\mathrm{and}\;\mathrm{reference}\;\mathrm{electrode}:2\mathrm{Li}{}_{0.25}\mathrm{FePO}{}_{4}+\mathrm{Li}{}^{+}+e{}^{-}\vec{\leftarrow }2\mathrm{Li}{}_{0.75}\mathrm{FePO}{}_{4}$$

In this cell design, the Li_0.25_FePO_4_ counter and reference electrode has enough capacity to provide all the electrons needed to sustain the electrochemical reactions at the working electrode, while maintaining a constant potential. This is achieved by employing Li_0.25_FePO_4_ electrodes with active material loading that is more than double of the active material loading of the LiFePO_4_ working electrode.

The validation of the cell design is presented in Figures S1 and S2 in the Supporting Information. Figure S1 shows that the results of the electrochemical characterisation of LiFePO_4_ composite electrodes in lithium half‐cells (i. e., when cycling against a lithium metal counter and reference electrode) are identical to the results obtained when cycling against the Li_0.25_FePO_4_ counter and reference electrode. These results were obtained in an organic carbonate electrolyte, LP57, to ensure the stability of the lithium metal electrode. Figure S1 clearly shows that the same electrochemical results are obtained with both types of counter electrodes, and that the only difference is that the potential scales are shifted, as expected. This is because, when cycling, the Li_0.25_FePO_4_ counter electrode is converted into Li_0.75_FePO_4_, and this is achieved at a constant potential of around 3.45 V vs. Li^+^/Li. Note that in this composition range of Li_0.25_FePO_4_ to Li_0.75_FePO_4_, the electrode is a mixture of two phases, LiFePO_4_ and FePO_4_, and consequently, the potential of the electrode is constant.[^34^, ^35^, ^36^]

Figure S2 compares the electrochemical performance of LiFePO_4_ cycled against the Li_0.25_FePO_4_ electrode in the organic LP57 electrolyte and in an aqueous 0.5 m Li_2_SO_4_ electrolyte. As expected, the same results are obtained, regardless of the electrolyte. Close inspection shows that the charging potential in LP57 electrolyte is a bit higher than in the aqueous electrolyte, which can be attributed to the lower conductivity of the former.

In conclusion, the cell design with LiFePO_4_ cycled against Li_0.25_FePO_4_ is a reliable approach to characterise the electrochemical behaviour of LiFePO_4_ electrodes, with the advantage that any degradation with cycling can be unambiguously ascribed to degradation processes of LiFePO_4_ materials, since the same degradation processes would affect the working and counter electrodes, because they are made of the same battery material. In the following, this advantageous cell design is used for the characterisation of the electrochemical behaviour and cycling stability of LiFePO_4_ in a variety of solution compositions.

### Lithium sequestration from the benchmark Li_2_SO_4_ solutions

First of all, the electrochemical behaviour of our chosen battery material for lithium sequestration, LiFePO_4_, was studied in the benchmark aqueous Li_2_SO_4_ electrolytes containing only lithium cations. The initial reaction of the electrochemical cell involves charging (oxidation) of the LiFePO_4_ working electrode (with release of lithium ions to the solution), and the subsequent discharge (reduction) is the reaction of lithium‐ion sequestration from solution into the FePO_4_ battery host. Thus, the electrochemical testing of the cell enables us to quantify the capacity for lithium‐ion absorption and release from the host material. The electrochemical characterisation of LiFePO_4_ was done with constant current cycling experiments at two different C‐rates. First, two charge/discharge cycles at C/10 were recorded (specific current=17 mA g^−1^), which were followed by five cycles at 1C (specific current=170 mA g^−1^). The potential limits were 0.3 and −0.3 V vs. Li_0.25_FePO_4_, which correspond to around 3.75 and 3.15 V vs. Li^+^/Li.

**Figure 2:**
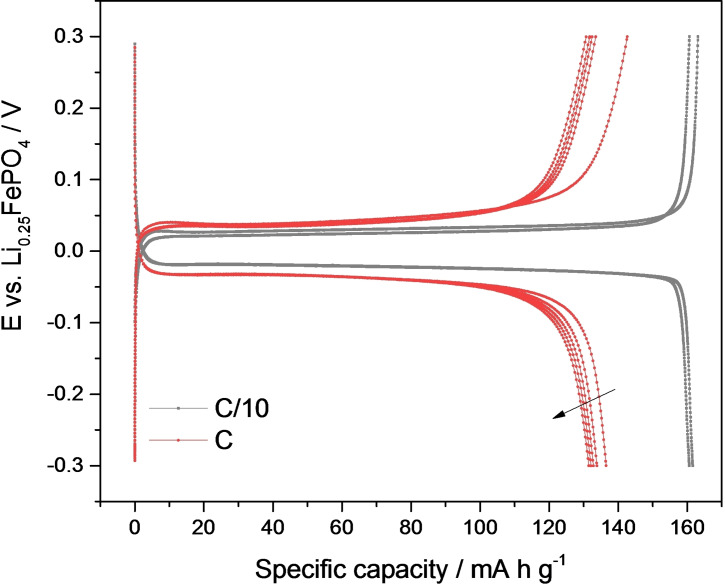
Charge/discharge profiles of LiFePO_4_ cycled against Li_0.25_FePO_4_ in 0.5 m Li_2_SO_4_ solution at C/10 (first 2 cycles, grey curves) and at 1C (subsequent 5 cycles, red curves).

Figure 2 shows an example of the electrochemical results obtained in 0.5 m Li_2_SO_4_ ([Li^+^]=1 m). The voltage profile shows the characteristic flat voltage plateaus, with low cell polarizations of the order of 20–25 mV, and with high charge/discharge capacity of 160 mA h g^−1^ at C/10 and 135 mA h g^−1^ at 1C, in good agreement with the best‐performance LiFePO_4_ electrodes reported in the literature.[^13^, ^37^, ^38^, ^39^, ^40^, ^41^, ^42^, ^43^, ^44^] The same high‐quality performance is obtained in lithium half cells (Figure S1).

**Figure 3:**
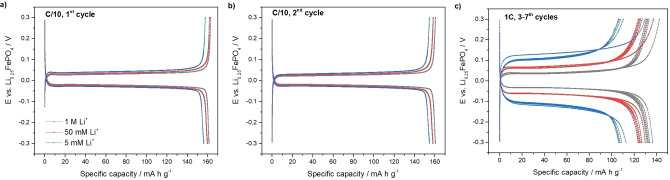
(a) 1st and (b) 2nd cycle charge/discharge profile recorded at C/10 and (c) subsequent five cycles at 1C, for LiFePO_4_ cycled against Li_0.25_FePO_4_ in aqueous Li_2_SO_4_ solutions with different lithium molarities: 1 m (grey curves), 50 mm (red curves) and 5 mm (blue curves).

Figure 3 compares the electrochemical behaviour of LiFePO_4_ electrodes in aqueous Li_2_SO_4_ solutions with different Li^+^ concentrations (1 m, 50 mm and 5 mm). The use of diluted Li_2_SO_4_ solutions enables the study of the reactions of lithium sequestration and release from the FePO_4_ host material at the lithium concentrations relevant in natural brine resources (see Table 1), but without the complications of the effects associated with the presence of other cations, which is studied in the next sections. At a C/10 rate, a slight reduction of the specific capacity was observed as the lithium concentration decreases, but even at the lowest Li^+^ concentration (5 mm), a high charge (oxidation) capacity of 156 mA h g^−1^ is obtained (corresponding to a mass of lithium inserted of 42 mg of lithium per g of FePO_4_), which demonstrates that the FePO_4_ host battery material is able to sequestrate a large amount of lithium via Equation (3) even when the Li^+^ concentration is very low, in agreement with previous studies^[17]^ (see Table S2:

(3) $$\mathrm{FePO}{}_{4}+\mathrm{Li}{}^{+}+e{}^{-}\to \mathrm{LiFePO}{}_{4}$$

**Table 1:** Molar concentrations of the different salts used to prepare the artificial brines.

Brine	[LiCl] [m]	[NaCl] [m]	[KCl] [m]	[MgCl_2_] [m]
Atacama	0.04	0.78	0.10	0.07
Olaroz	0.18	5.00	0.28	–
Central Altiplano	0.06	4.00	0.20	0.30

When the measurements are done at a higher C‐rate of 1C, the performance of LiFePO_4_ electrodes is still very good, even at low lithium concentrations, as shown in Figure 3. A high capacity of around 110 mA h g^−1^ is obtained at the lowest lithium concentration (5 mm), which corresponds to an amount of lithium inserted of 30 mg of lithium per g of FePO_4_. The results in Figure 3 also show that these faster experiments of 1C cycling also produce a small increase in cell voltage polarization. Decreasing the Li^+^ concentration from 1 m to 5 mm produces a 70 mV shift in the voltage plateaus values, which can be attributed to the decrease in solution conductivity.

Overall, the experiments in the benchmark Li_2_SO_4_ solutions demonstrate that FePO_4_ can be used as a host battery material for lithium‐ion sequestration even for low Li^+^ concentrations of 5 mm, producing high lithium absorption capacities equivalent to 42 and 30 mg_Li+_g_FePO4_^−1^, in constant current experiments at C/10 and 1C rates. The slight decrease in the lithium uptake at high current densities is in agreement with previous studies^[18]^ (see Table S2).

### Lithium sequestration from solutions containing an excess of other ions (Na^+^, K^+^ and Mg^2+^)

Natural lithium brines contain lithium in a concentration that is much lower than other cations (called “co‐cations”) such as sodium, potassium and magnesium. Consequently, the suitability of the use of FePO_4_ as host sequestration material critically depends on its ability to selectively sequestrate only lithium ions in the presence of an excess of concentration of other cations.

**Figure 4:**
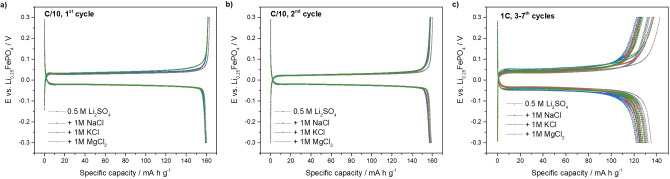
As in Figure 3 but with aqueous 0.5 m Li_2_SO_4_ solution (grey curves) and with 0.5 m Li_2_SO_4_ solution containing either 1 m NaCl (red curves), 1 m KCl (blue curves) or 1 m MgCl_2_ (green curves).

The influence of other cations (Na^+^, K^+^ and Mg^2+^) on the electrochemical capacity of lithium absorption/release by FePO_4_ electrodes was studied in aqueous 0.5 m Li_2_SO_4_ solution (1 m Li^+^) in the presence of 1 m concentrations of Na^+^, K^+^ or Mg^2+^ (0.5 m Li_2_SO_4_+1 m NaCl, 0.5 m Li_2_SO_4_+1 m KCl or 0.5 m Li_2_SO_4_+1 m MgCl_2_, respectively). Typical constant‐current charge/discharge curves of LiFePO_4_ are shown in Figure 4, demonstrating that the addition of Na^+^, K^+^ or Mg^2+^, in the same concentration as Li^+^, does not produce any significant change in the electrochemical results, thus demonstrating that none of these cations are inserted into the FePO_4_ battery material under the current experimental conditions.

The high lithium selectivity of the FePO_4_ host battery material is in agreement with previous studies reporting a high lithium purity (90–96 %) of the LiFePO_4_ reaction product obtained in brines.[^13^, ^14^] Electrochemical measurements of the FePO_4_ host material in solutions containing Li^+^ and Na^+^ showed that Na^+^ insertion into FePO_4_ is kinetically and thermodynamically less favourable than that of Li^+^,[^17^, ^45^, ^46^, ^47^] and in constant‐current experiments, it takes place at potentials that are around 0.15 V more negative than those of Li^+^.[^17^, ^47^] The insertion of Mg^2+^ or K^+^ into FePO_4_ is even more sluggish than that of Na^+^,[^46^, ^47^, ^48^] and in constant‐current experiments, it takes place at potentials that are around 0.2–0.3 V more negative than those of Li^+^.^[47]^ Consequently, the insertion of Na^+^, K^+^ or Mg^2+^ into FePO_4_ is not expected for the potential range employed here, but it is remarkable that the capacity of Li^+^ insertion into FePO_4_ is not affected by any of these co‐cations. The next section investigates the ability of the FePO_4_ host material to selectively sequestrate Li^+^ from solutions with composition mimicking natural lithium brines.

### Lithium sequestration from artificial brines

In order to evaluate the suitability of the use of FePO_4_ host material for the production of lithium from natural lithium brines, the electrochemical behaviour of LiFePO_4_ was studied, in the new cell design developed here, using aqueous electrolytes with composition simulating the natural lithium resources in Atacama (Chile),[^29^, ^30^] Olaroz (Argentina)[^24^, ^31^, ^32^] and in Central Altiplano (Bolivia)^[33]^ (see Table 1). These artificial brines contain sodium, magnesium and potassium in higher concentration than lithium.

**Figure 5:**
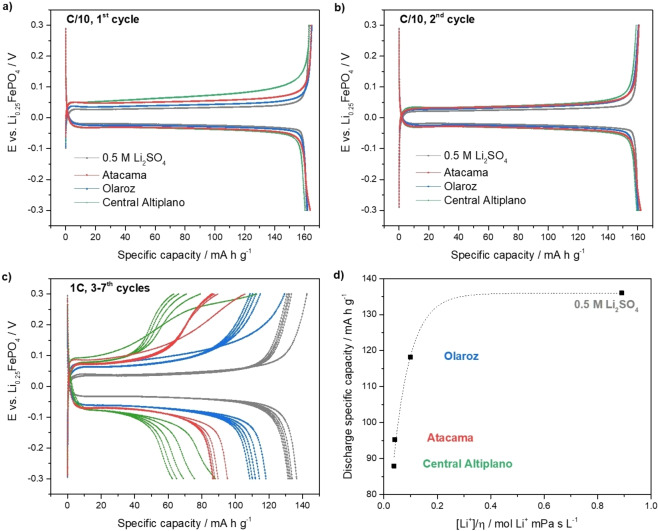
As in Figure 3 but with aqueous 0.5 m Li_2_SO_4_ solution (grey curves) and with the synthetic brines: Atacama (red curves), Olaroz (blue curves) and Central Altiplano (green curves). (d) Correlation of the first discharge specific capacity at 1C and the ratio of the lithium concentration and viscosity. The composition of the synthetic brines is shown in Table 1.

Figure 5 shows a typical example of the constant‐current cycling of LiFePO_4_ electrodes in the selected brines and the benchmark 0.5 m Li_2_SO_4_ solution. At C/10, a high insertion capacity of around 160 mA h g^−1^ is obtained in all cases, which corresponds to the sequestration of 43 mg of lithium per g of FePO_4_. The lithium uptake is comparable, but somewhat higher, than in previous studies[^19^, ^45^, ^47^, ^48^] (see Table S2). In addition, the same electrochemical behaviour is observed in all brines, which indicates that the electrochemical reactions only involve Li^+^, and the absorption of other co‐cations is quantitatively excluded. Further evidence of the high lithium selectivity is presented below using XRD measurements. The high cell‐to‐cell reproducibility of these measurements is shown in Figure S3, and Figure S4 shows that more cell‐to‐cell variability was obtained with the highly viscous Central Altiplano brine, which can be tentatively attributed to non‐ideal, manual cell assembly.

For the experiments done at a faster C‐rate of 1C, the capacity in the brines (88–118 mA h g^−1^) is smaller than in the benchmark 0.5 m Li_2_SO_4_ solution (136 mA h g^−1^), as expected due to the lower Li^+^ concentration in the brines. However, the Li^+^ concentration is not the only factor affecting the high‐rate capacities: for example, the Li^+^ concentration in the Atacama brine is lower than in the Central Altiplano brine, but the capacity is higher. These effects can be attributed to the presence of cations other than Li^+^. Indeed, previous studies have suggested that the co‐adsorption of other cations (Na^+^, Mg^2+^ and/or K^+^) at the surface of the electrode might lead to partial blocking of the Li^+^ insertion channels, thus decreasing the rate of Li^+^ insertion.[^31^, ^32^, ^49^] Here we have found that the viscosity of the solution is a key determining factor of rate of lithium insertion. Figure 5d shows that there is a quantitative correlation between the experimental capacity at 1C and the ratio of the Li^+^ concentration and the dynamic viscosity (*η*) of the solution (see Table 2). This correlation shows that the capacities achievable at high C‐rates, under the current experimental conditions, depend on the rate of Li^+^ transport in the brines, which in turn depends on the product of the Li^+^ concentration and the Li^+^ diffusion coefficient (and where the latter decreases as the solution viscosity increases).^[50]^

**Table 2:** Properties of the benchmark electrolyte (0.5 m Li_2_SO_4_) and the synthetic brines studied in this work.

Brine	[Li^+^] [m]	[Na^+^]/[Li^+^]	[K^+^]/[Li^+^]	[Mg^2+^]/[Li^+^]	*η* [mPa s]	[Li^+^]/*η* [m mPa^−1^ s^−1^]
0.5 m Li_2_SO_4_	1.00	–	–	–	1.13	0.89
Atacama	0.04	19.5	2.50	1.75	0.97	0.041
Olaroz	0.18	27.8	1.56	–	1.79	0.10
Central Altiplano	0.06	66.7	3.33	5.00	1.60	0.038

The effect of the presence of cations other than Li^+^ in the brines was studied further using modifications of the Olaroz brine, which had the highest viscosity (Table 2). The composition and properties of the brine modifications are shown in Tables S3–S4 and the electrochemical results in Figures S7–S9. It is found that the addition of Mg^2+^ decreases the rate of lithium insertion, and the removal of Na^+^ increases it, in agreement with previous findings of the detrimental effect of the presence of ions other than Li^+^.[^31^, ^32^, ^49^] Interestingly, the quantitative correlation between the experimental, high‐rate capacity, at 1C rate, and the ratio of lithium concentration and viscosity (Figure S8) holds for all the brine compositions here studied, confirming that the brine viscosity is a key determining factor.

We also explored a new method to enhance the kinetics of the lithium sequestration process, based on brine dilution (Figure S7), which produces a dramatic increase of the high‐rate capacity, at 1C rate, from 83 mA h g^−1^ prior to dilution to 110 mA h g^−1^ after dilution (Table S3). Although the improvement was unexpected, since dilution decreases the Li^+^ concentration, we find that the approach of brine dilution also enhances the cycling stability (see below), and thus, it is identified as a highly promising approach for the exploitation of concentrated brines.

In summary, the combination of the low Li^+^ concentration and the presence of cations other than lithium in the brines slows down the kinetics of Li^+^ insertion into FePO_4_, and consequently, the lithium absorption capacity at high currents. Fortunately, these capacity limitations are efficiently removed by the simple dilution of the brine with water, which appears as a highly promising new strategy for the production of lithium using battery materials. In the following section, XRD analysis of the cycled electrodes is used to evaluate to the selectivity of the FePO_4_ host structure to the absorption of only lithium ions.

### Selectivity of lithium sequestration from artificial brines

**Figure 6:**
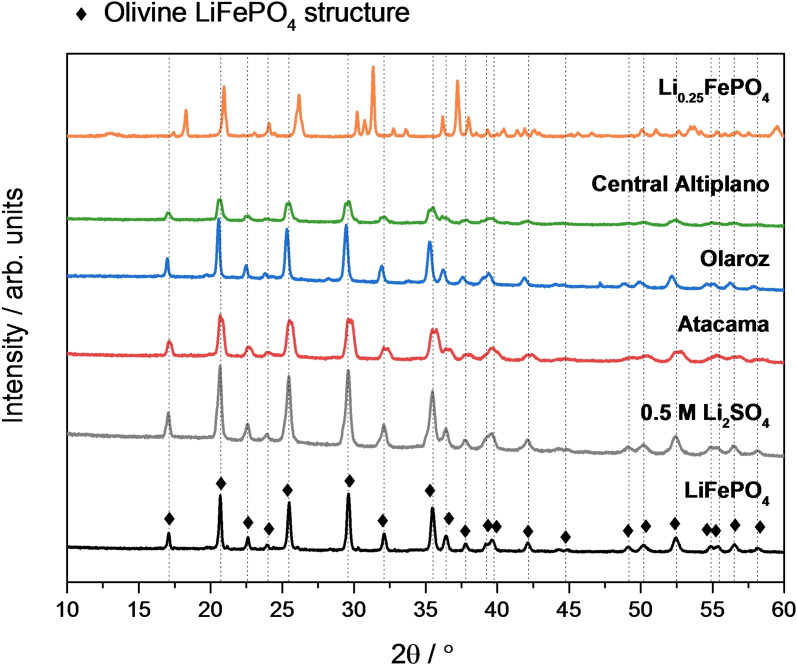
XRD patterns of the LiFePO_4_ electrodes after one charge/discharge cycle at C/10 in 0.5 m Li_2_SO_4_ and in the different brines. The diffractograms of pristine (i. e., uncycled) LiFePO_4_ and Li_0.25_FePO_4_ electrodes also shown for comparison. The vertical lines mark the positions of the characteristic peaks due to LiFePO_4_, showing that the electrodes cycled in 0.5 m Li_2_SO_4_ solution and in the different brines only show the peaks characteristic of LiFePO_4_. Differences in diffraction peak intensities are due to small changes in electrode alignment, and do not indicate changes in the crystallinity of LiFePO_4_ in the electrodes

XRD analysis of the cycled LiFePO_4_ electrodes was done in order to assess the selectivity of lithium sequestration. For that purpose, after one full charge and discharge cycle of the LiFePO_4_ electrodes in the electrochemical cell, which is expected to induce the oxidation of LiFePO_4_ to FePO_4_ and then the subsequent reduction back to LiFePO_4_, the LiFePO_4_ electrodes were extracted from the cell and characterized by XRD, and the results are shown in Figure 6. For comparison purposes, Figure 6 also includes the XRD patterns of the pristine LiFePO_4_ and Li_0.25_FePO_4_ electrodes, prior to any electrochemical cycling.

The results in Figure 6 show that the LiFePO_4_ electrodes cycled in the electrochemical cell, with the artificial brines or with the 0.5 m Li_2_SO_4_ benchmark solution, exhibit the characteristic diffractogram of the olivine LiFePO_4_ structure. No other peaks appear in the diffraction patterns, and all the peaks expected for LiFePO_4_ can be identified, thus indicating that only lithium ions have been inserted in the host FePO_4_ structure. The insertion of Na^+^ into FePO_4_ would result in characteristic diffraction peaks associated to olivine NaFePO_4_,^[51]^ which are not observed in Figure 6. The intercalation of K^+^ or Mg^2+^ into amorphous FePO_4_ structure has also been reported to produce the appearance of diffraction peaks at different reflection positions, and therefore, it can be excluded too.[^52^, ^53^, ^54^] In addition, fully lithiated LiFePO_4_ electrodes were obtained in all the cases since the characteristic diffraction peaks of FePO_4_ were not observed by XRD.[^13^, ^14^] As a comparison, the presence of FePO_4_ characteristic peaks can be clearly observed in the diffractrogram of the Li_0.25_FePO_4_ electrode, which contains a mixture of the LiFePO_4_ and FePO_4_ phases.

Previous works have also reported very high lithium selectivity of the FePO_4_ material. The elemental analysis of LiFePO_4_ produced from brines show high lithium purity (90–96 %),[^13^, ^14^] and high lithium purity of the recovery solutions using LiFePO_4_ electrodes obtained from brines has also been reported[^17^, ^18^, ^28^] (see Table S2). High lithium selectivity has also been reported for the capture of lithium from seawater by a polydopamine‐coated FePO_4_ electrode, producing a Li/Na molar ratio of 43.3, corresponding to a lithium purity of 98 %.^[55]^ On the other hand, a previous study reported the insertion of Mg^2+^ in FePO_4_,^[48]^ but the results were obtained in a brine with very high ratio of Mg^2+^ to Li^+^ concentrations, and in experiments applying a large voltage difference of 1 V between FePO_4_ and LiFePO_4_ electrodes; the electrochemical results in Figure 5 suggests that the insertion of only Li^+^ ions in the FePO_4_ structure can be achieved by avoiding polarisation to potentials lower than 0.3 V vs Li_0.25_FePO_4_. Finally, the adsorption of co‐cations (specifically, Na^+^) on LiMn_2_O_4_ electrodes in brines has been detected in previous studies using X‐ray photoelectron spectroscopy (XPS) measurements;[^31^, ^49^] however, if co‐cation adsorption also took place on LiFePO_4_, this would not significantly affect the purity of the LiFePO_4_ reaction product nor of the recovery solutions, since the adsorption process is confined to the surface.

In summary, the XRD analysis of the cycled electrodes indicates that full and selective lithium insertion is achieved into the host FePO_4_ structure. In the next sections, the cycling stability and preservation of the lithium selectivity is investigated.

### Cycling stability of FePO_4_ electrodes in artificial brines

**Figure 7:**
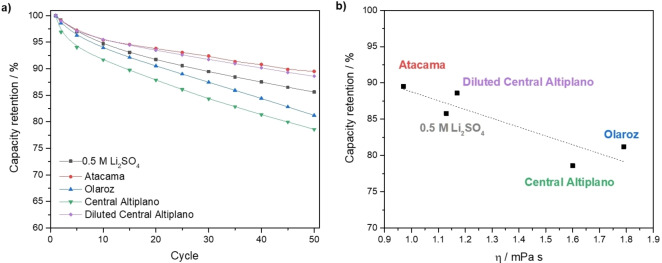
(a) Capacity retention against cycle number for LiFePO_4_ electrodes cycled against Li_0.25_FePO_4_ at C/10 in aqueous 0.5 m Li_2_SO_4_ solution and synthetic brines. (b) Percentage of capacity retention at cycle 50 as a function of the brine viscosity. The composition of the synthetic brines is shown in Table 1, and additional measurements were done with Central Altiplano brine diluted by three times with water (2 mL of water were added per mL of brine).

The cycling stability of LiFePO_4_ electrodes in the lithium brines was studied for 50 cycles at C/10. Figure 7a shows the capacity decay with cycling in the benchmark electrolyte, 0.5 m Li_2_SO_4_, and in the lithium brines. Good cycling stability was observed in the Li_2_SO_4_ solution and the less viscous brine (Atacama), with capacity retention of 86 and 90 %, respectively. In the more viscous brines, a slightly higher capacity fading was observed, of 81 and 79 % for Olaroz and Central Altiplano, respectively. The high cell‐to‐cell reproducibility of these measurements is shown in Figures S5 and S6. Figure 7b shows that the capacity retention is correlated with the solution viscosity.

The capacity retention with cycling found here is superior, or comparable, to previously reported values for the use of the LiFePO_4_ for lithium sequestration from brines. Trócoli et al.^[25]^ reported a capacity retention of 21 % after 25 cycles at 1C in the Atacama brine and He et al.^[47]^ reported a capacity retention of 84 % after 50 cycles at 0.3 mA cm^−2^ (equivalent to a C‐rate of ≈C/23) in a brine composition with a relatively high lithium concentration of 2 g L^−1^ (equivalent to ≈0.3 m). These differences in behaviour can be ascribed to the effect of the counter electrode material and/or to the properties (particle size, carbon coating, etc.) of the LiFePO_4_, thus evidencing that much further work is needed for the investigation of the use of battery materials for lithium sequestration from brines.

It is also important to note that the capacity retention with cycling found here is in good agreement with the best‐performing aqueous LiFePO_4_ cells reported in the literature. Xia and co‐workers^[56]^ reported an excellent capacity retention of 90 % after 1000 cycles at 6C with LiFePO_4_ vs. LiTi_2_(PO4)_3_ cells, but the slower cycling at C/8 produced a more moderate capacity retention of 85 % after 50 cycles, in good agreement with the present results. The faster capacity fade at lower C‐rates was explained by the fact that degradation processes are time dependent, rather than cycle number dependent, as it is found in non‐aqueous Li‐ion cells.[^57^, ^58^] Zhao et al.^[59]^ reported LiFePO_4_ vs. LiV_3_O_8_ cells with 80 % capacity retention after 100 cycles at 1C with 2 m LiNO_3_ electrolyte, and with no capacity fading using 9 m LiNO_3_ electrolyte. Gordon et al.^[60]^ reported LiFePO_4_ vs Li_0.47_FePO_4_ cells with 40 % capacity retention after 500 cycles at 1.1C with 0.5 m Li_2_SO_4_ electrolyte, and 80 % capacity retention using saturated LiNO_3_ electrolyte. Taking into account the slower C‐rate of C/10 used here, which increases the testing time and thus degradation processes, the capacity retention with cycling found here is comparable or better than in previous studies. This is quite remarkable since the use of brine solutions, containing high concentrations of NaCl, MgCl_2_ and KCl, and low concentrations of lithium cations, can potentially induce more severe degradation. Indeed, by testing the different brine compositions under exactly the same experimental conditions, we can clearly demonstrate that the more concentrated brines (Olaroz and Central Altiplano) enhance capacity degradation.

**Figure 8:**
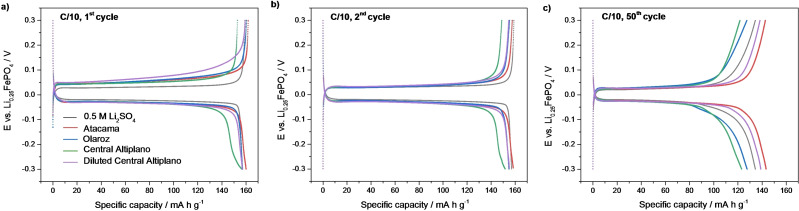
(a) 1st, (b) 2nd and (c) 50th cycle charge/discharge profile for LiFePO_4_ electrodes cycled against Li_0.25_FePO_4_ at C/10 in aqueous 0.5 m Li_2_SO_4_ solution and synthetic brines, as in Figure 6. The results obtained in the Central Altiplano brine show slightly less capacity than in Figure 4, due to the higher cell‐to‐cell variability that occurred with this highly viscous brine, ascribable to the difficulty of optimal cell assembly.

Another very significant result from this work is that we found that degradation of capacity with cycling can be significantly suppressed by simply diluting the brine with water. This is illustrated for the case of the brine with highest capacity fade (Central Altiplano): dilution of the brine with water in a 1 : 2 brine/water volume ratio produces a significant improvement in capacity retention, which is surprising since the dilution not only decreases the viscosity but also the concentration of lithium ions. However, the dilution of the brine does not produce any significant decrease in capacity or potential polarization, as shown in the 1st and 2nd cycles charge/discharge profile in Figure 8a, b. Indeed, it is observed that the dilution of the brine only produces the beneficial effect of decreasing the capacity fade with cycling: Figure 8c shows that at the 50th cycle, a much‐increased capacity is obtained in the diluted brine, with respect to the brine without dilution.

In addition, the fact that the shape of the 50th cycle charge/discharge profiles is very similar to that of the 1st and 2nd cycles, suggests that the high lithium selectivity is retained with cycling, since the process of insertion of other ions into the FePO_4_ structure would produce extra capacity contributions at different potential values.

In the next section, XRD, Raman spectroscopy and SEM characterisation of the electrodes after cycling is used to investigate the retention of the lithium selectivity and the causes of capacity fade with cycling.

### Post‐mortem analysis of cycled LiFePO_4_ electrodes

In order to investigate the causes of capacity fade of the LiFePO_4_ electrodes, the cells were disassembled after 50 charge/discharge cycles at C/10, in which the electrochemical test was finished with the fully discharged (lithiated) LiFePO_4_ electrode, and the electrodes were characterized by XRD, Raman spectroscopy and SEM.

**Figure 9:**
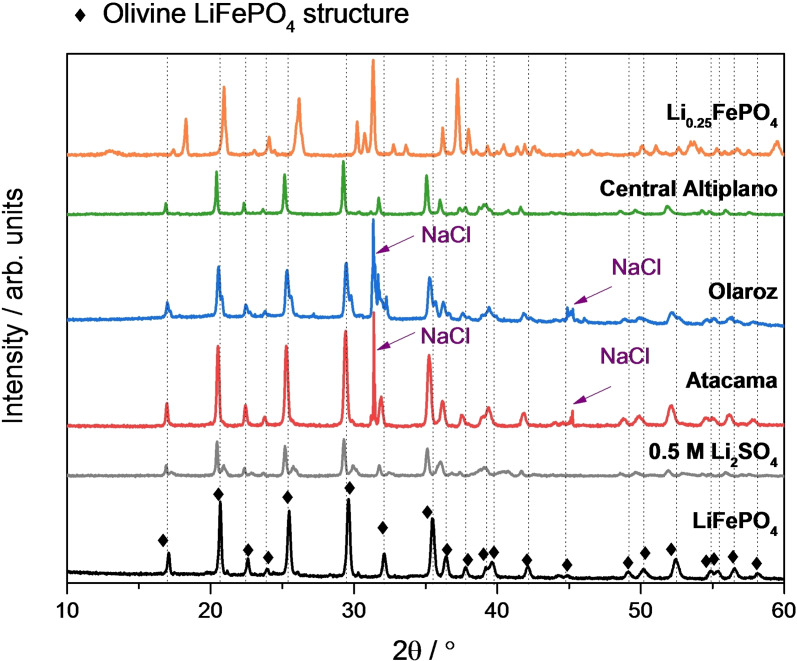
As Figure 6, except that the electrodes were cycled for 50 charge/discharge cycles. In some of the diffractograms, the presence of diffraction peaks corresponding to NaCl salt precipitate is seen, which is due to the partial evaporation of the brine impregnated in the electrode after the cell disassembly.

Figure 9 shows the results of the XRD characterisation, showing that all the electrodes cycled in the 0.5 m Li_2_SO_4_ solution or brine show the characteristic pattern of LiFePO_4_. This confirms that the selectivity towards lithium‐ion insertion is retained over cycling, and the insertion of other ions (Na^+^, Mg^2+^ or K^+^) is prevented due to the high selectivity of the LiFePO_4_ material. The good agreement with the characteristic XRD pattern of LiFePO_4_ also indicates that the LiFePO_4_ is well preserved during cycling. In some cases, some extra diffraction peaks are observed, which can be ascribed to the presence of solid NaCl deposits. After cell disassembly, the electrodes contain a volume of impregnated brine solution, and during characterisation, the evaporation of water from the brine produces the precipitation of the brine salt components, which are primarily NaCl.

**Figure 10:**
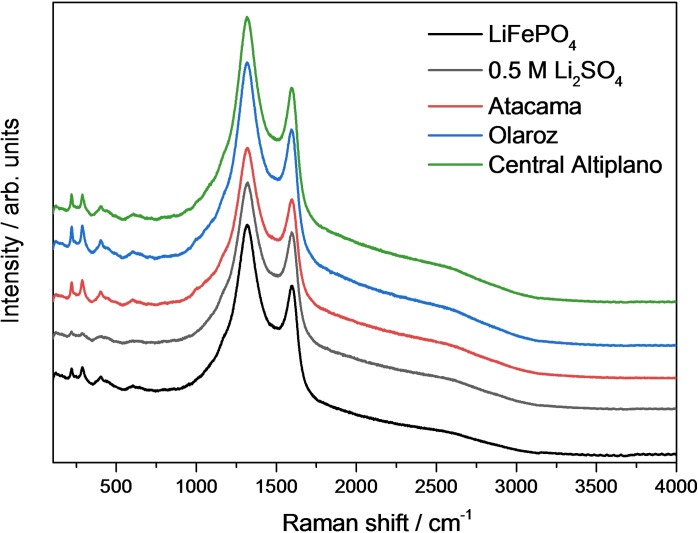
Raman spectra of the LiFePO_4_ electrodes after 50 charge/discharge cycles at C/10 in 0.5 m Li_2_SO_4_ and in the different brines. The spectrum of the pristine (i. e., uncycled) LiFePO_4_ electrode is also shown for comparison.

Figure 10 compares the Raman spectra of the pristine and cycled electrodes, showing, again, no visible difference between the spectra. In all cases, two prominent peaks at around 1320 and 1600 cm^−1^ are seen, which are ascribed to the D and G bands of amorphous carbon,[^61^, ^62^] present in the electrodes as conductive additive and in the form of carbon coating of the LiFePO_4_ particles. Previous studies[^54^, ^63^, ^64^, ^65^, ^66^] have also shown that the Raman spectra of carbon‐coated LiFePO_4_ is dominated by the bands of amorphous carbon, thus indicating that the carbon coating is homogeneous and pinhole free. Accordingly, the fact that the Raman spectra of the LiFePO_4_ electrodes is not altered after 50 cycles of charge and discharge, indicates that the carbon coating does not undergo degradation. Indeed, it has been shown by mass spectrometry measurements that the corrosion of carbon takes place at much higher potentials than those used here.^[63]^

**Figure 11:**
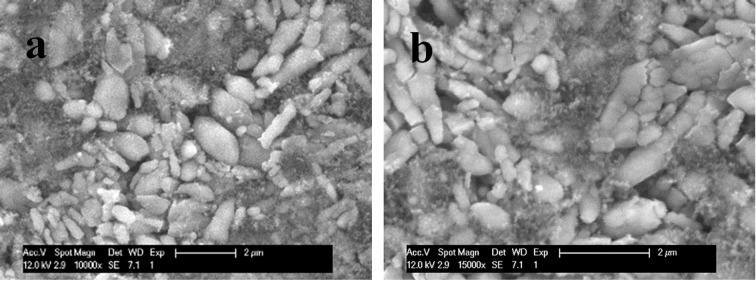
SEM images of (a) the pristine (i. e., uncycled) LiFePO_4_ electrode and (b) the LiFePO_4_ electrode after 50 charge/discharge cycles at C/10 in the Central Altiplano brine.

SEM images of the surface of the pristine (i. e., uncycled) LiFePO_4_ electrode and the electrode cycled in Central Altiplano brine are compared in Figure 11. It is seen that the electrochemical cycling induced the formation of cracks in the carbon‐coated LiFePO_4_ particles in the electrode, which can be ascribed to the repetitive expansion/contraction of the particles during lithium insertion/extraction.[^34^, ^36^] The formation of cracks can exacerbate the effects of degradation reactions taking place at the particle‐electrolyte interphase, thus leading to capacity fading, as pointed out previously,[^60^, ^67^, ^68^, ^69^] and consequently, smaller particle sizes or alternative particle morphologies (e. g., nanoplates) could significantly improve the cycling stability.

In view of the SEM images of the cycled electrodes in Figure 11, particle cracking is identified as a likely cause of capacity fading. Indeed, previous studies have reported that LiFePO_4_ slowly degrades in contact with water.[^70^, ^71^, ^72^, ^73^, ^74^, ^75^, ^76^] Porcher et al.^[74]^ found that the immersion of LiFePO_4_ particles in water produced the formation of a Li_3_PO_4_ surface coating on the LiFePO_4_ particles as well as Fe dissolution. Yu et al.^[75]^ showed that the presence of Li_3_PO_4_ or other impurities, in contact with water, can act synergistically to promote Fe dissolution, and thus deteriorate the LiFePO_4_ electrochemical performance. Although the presence of a carbon coating mitigates these undesirable reactions, it does not provide full protection.^[72]^

The deleterious interfacial reactions between the LiFePO_4_ particles and the aqueous brines are exacerbated with particle cracking since the latter increases the surface of LiFePO_4_ exposed to the brines. In addition, Figure 7b shows that increasing the brine viscosity exacerbates the capacity fade with cycling. Therefore, we propose that the slow rate of Li^+^ transport in the highly viscous brines causes more inhomogeneity of the Li^+^ insertion reaction in the LiFePO_4_ particle, and differences in Li^+^ concentration inside LiFePO_4_ particles are known to facilitate particle cracking.[^77^, ^78^, ^79^, ^80^, ^81^] This hypothesis explains why brine dilution mitigates capacity fading, via the enhanced rate of Li^+^ transport achieved by decreasing viscosity. Simultaneously, the decreased viscosity achieved by dilution also enhances the rate capability, as shown in Figure S7c, which is also ascribed to the enhanced rate of Li^+^ transport.

Finally, it is also important to note that the LiFePO_4_ employed in this work has been optimized for use in non‐aqueous Li‐ion batteries, and thus, further improvements in the long‐term cycling stability can be achieved by material development, by for example, employing different coating materials.[^82^, ^83^, ^84^, ^85^] It is also important to note that the results here presented were obtained in the presence of O_2_ dissolved in the electrolyte (since all components were exposed to air and cell assembly was done in air), and previous studies has shown that O_2_ accelerates LiFePO_4_ degradation.[^70^, ^73^] Therefore, further performance improvements can also be expected by removing O_2_ from the cells.

## Conclusion

The cycling stability and lithium selectivity of LiFePO_4_ as a host material for lithium sequestration from brines is investigated here by combining electrochemical measurements in “nearly‐symmetrical” cells (made with a Li_0.25_FePO_4_ counter and reference electrode) and X‐ray diffraction (XRD), Raman spectroscopy and scanning electron microscopy (SEM) characterisation of the pristine and cycled electrodes. After the validation of the cell design with benchmark Li_2_SO_4_ electrolyte solutions, the study was extended to three selected brine compositions that mimic strategic lithium brine resources in Chile, Bolivia and Argentina.

For all the brine compositions, a high lithium insertion capacity of around 160 mA h g^−1^ is obtained in constant‐current experiments at C/10. At a faster C‐rate of 1C, the lithium insertion capacity depends on the brine composition, and specifically, it is found to correlate with the ratio of lithium concentration and solution viscosity. Moreover, the dilution of highly concentrated brines with water is found to be a new and effective strategy to raise the rate capability, producing an increase in the capacity at 1C from 83 mA h g^−1^ (without dilution) to 110 mA h g^−1^ (with dilution).

The electrochemical results also evidence very high lithium selectivity of the FePO_4_ host material, which is confirmed by XRD measurements of the cycled electrodes, showing the characteristic diffraction peaks of olivine LiFePO_4_ and the absence of any other peaks associated with the insertion of other ions (Na^+^, Mg^2+^ or K^+^). Raman measurements show that the carbon coating of the LiFePO_4_ is well preserved with cycling. On the other hand, SEM images revealed the formation of cracks in LiFePO_4_ particles in the surface of cycled electrodes, which appears to be an important cause of capacity fading, and thus alternative LiFePO_4_ morphologies and sizes could produce better results.

The cycling stability of LiFePO_4_ in the brine solutions is better or comparable to previously reported in brine solutions and in aqueous Li‐ion cells. After 50 cycles at C/10 (where the low C‐rate is chosen to investigate the effect of sluggish degradation processes), the capacity retention ranges from 90 to 79 %, depending on the brine composition, and with more viscous brines producing more degradation. Interestingly, we report for the first time that a simple dilution of the brines with water produces a significant improvement in the capacity retention: from 79 (without dilution) to 89 % (with dilution) after 50 cycles.

Finally, it is important to highlight that the LiFePO_4_ material employed here has been designed for non‐aqueous lithium‐ion batteries. Thus, much further improvements in capacity retention could be achieved by the development of surface coatings specifically designed to mitigate degradation reaction in the brine solutions, which clearly deserve further study.

## Experimental Section

### Chemical delithiation of lithium iron phosphate

Commercial LiFePO_4_ (provided by Tatung, battery grade) was chemically delithiated using K_2_S_2_O_8_ (Sigma Aldrich, ACS grade, ≥99 %) as an oxidizing agent following a reported procedure.[^13^, ^14^, ^86^] Briefly, K_2_S_2_O_8_ (0.1 m) and LiFePO_4_ (0.2 m) were mixed in ultrapure water and the reaction was held for 24 h at room temperature under continuous stirring. The resultant powder was filtered, washed and dried overnight at 80 °C.

### Preparation of LiFePO_4_ and Li_0.25_FePO_4_ electrodes

LiFePO_4_ and Li_0.25_FePO_4_ were used as working and counter electrodes, respectively. LiFePO_4_ electrodes were prepared with LiFePO_4_ (Tatung, as received), whereas in the case of Li_0.25_FePO_4_ a mixture of LiFePO_4_ (Tatung, as received) and FePO_4_ (obtained by chemical delithiation of LiFePO_4_) in a weight ratio of 1 : 3 was used as the active material. LiFePO_4_ and Li_0.25_FePO_4_ electrodes were prepared by mixing the active material, carbon black (Timcal SUPER C65) and polyvinylidene fluoride (PVDF, Solef® 5130) in a weight proportion of 8 : 1 : 1, respectively, and using *N*‐methyl‐2‐pyrrolidone (NMP) as solvent, with a volume of 4 mL of NMP per g of solid. The resultant slurry was mixed at 2000 rpm for 5 min with a planetary mixer (THINKY ARE‐250), with three repetitions of the mixing process. Subsequently, the slurry was hand coated on a titanium foil (Advent Research Materials, thickness=0.025 mm, 99.6 %) with a K‐bar to a wet thickness of 200 μm, for both LiFePO_4_ and Li_0.25_FePO_4_ formulations. The coated electrode sheet was dried in a vacuum oven at 80 °C overnight. In the case of Li_0.25_FePO_4_, a consecutive layer of 200 μm of a fresh slurry was deposited and dried again overnight. Once dried, the coated foil was punched into discs (diameter=11 mm, Nogami hand‐held precision punch). Finally, LiFePO_4_ working electrodes and Li_0.25_FePO_4_ counter electrodes were obtained by calendaring the disks to 10 or 2 tonne with a Specac hydraulic press, respectively. The active material mass loading was around 3 mg cm^−2^ for LiFePO_4_ and 6–7 mg cm^−2^ for Li_0.25_FePO_4_.

### Electrochemical extraction of lithium from artificial brines in LiFePO_4_/Li_0.25_FePO_4_ cells

Electrochemical extraction of lithium was performed in three different artificial brines. The molar compositions are listed in Table 2. Chloride salts, LiCl, NaCl, KCl and MgCl_2_ ⋅ 6H_2_O (Sigma Aldrich, ACS grade, ≥99 %), were used for the preparation of the brines. These artificial brines represent typical compositions in the lithium reserves in Atacama (Chile),[^29^, ^30^] Olaroz (Argentina)[^24^, ^31^, ^32^] and in Central Altiplano (Bolivia).^[33]^

The electrochemical tests were performed in PFA Swagelok® type cells, using the LiFePO_4_ electrodes as the working electrode and Li_0.25_FePO_4_ electrodes as both counter and reference electrode. Titanium current collectors were used for both electrodes. The cell also contained two glass fibre separators (Whatman®, grade GF‐F, 12 mm diameter) soaked with 150 μL of the artificial brines or aqueous Li_2_SO_4_ solutions. The cells were assembled in air and left to rest for 10 h before the electrochemical measurements.

Galvanostatic cycling with potential limitations (GCPL) were carried out with the cells placed in a Memmert climatic chamber set at 25 °C, using a VMP2 multichannel potentiostat (BioLogic) at C‐rates of C/10 and 1C with potential limits of 0.3 and −0.3 V vs. Li_0.25_FePO_4_ (corresponding to 3.75 and 3.15 V vs. Li^+^/Li). C‐rates were calculated from the theoretical specific capacity of LiFePO_4_ (170 mA h g^−1^), that is, a C‐rate of C/10 corresponds to a specific current (normalized by the mass of the LiFePO_4_ active material) of 17 mA g^−1^.

### XRD, Raman and SEM characterisation

Ex‐situ XRD patterns of the electrodes were collected in grazing incidence geometry (1° incidence angle) with Cu‐K_α_ radiation (*λ*=1.5418 Å) using a Rigaku SmartLab diffractometer with Hypix 2D detector. Scans were performed at 1° min^−1^ for 2*θ* values from 10 to 60°.

Ex‐situ Raman spectra of the electrodes were recorded on a Renishaw inVia spectrometer (excitation wavelength 783 nm, absolute power≥300 mV) with a Leica DM 25,000 M microscope, ×50 objective lens providing a 5 μm spot size on the sample. Extended spectra (Raman shift 100–4000 cm^−1^) were obtained at 1 % laser power, 10 s exposure time for 10 accumulations. WiRE 4.1 software was used to process the data.

SEM images were acquired with a Philips XL30 Environmental Scanning Electron Microscope.

### Measurement of the viscosity of the brines

The viscosity of the different brines was calculated from the time the brine required to flow between two marks in a 50 Cannon‐Fenske viscometer tube (Sigma‐Aldrich), which is usually called the “efflux time”. The density of the solutions was obtained by measuring the mass of solution contained in a 25 mL volumetric flask. The measurements were done at 25 °C (Table 3).

**Table 3:** Viscosity and density of the studied solutions at 25 °C.

Solution	Efflux time [s]	Kinematic viscosity [mm^2^ s^−1^]	Density [g mL^−1^]	Dynamic viscosity [mPa s]
0.5 m Li_2_SO_4_	335	1.08	1.04	1.13
Atacama	290	0.94	1.03	0.97
Olaroz	469	1.52	1.18	1.79
Central Altiplano	426	1.39	1.16	1.60

The kinematic viscosity is given by the product of the efflux time times and the viscometer constant (in this case, 0.003240±0.000011 mm^2^ s^−2^). The dynamic viscosity equals the kinematic viscosity times the density of the solution.

## Conflict of interest

The authors declare no conflict of interest.

## Supporting information

As a service to our authors and readers, this journal provides supporting information supplied by the authors. Such materials are peer reviewed and may be re‐organized for online delivery, but are not copy‐edited or typeset. Technical support issues arising from supporting information (other than missing files) should be addressed to the authors.

Supporting Information
