# Supplementary material for: LiFePO4 Battery Material for the Production of Lithium from Brines: Effect of Brine Composition and Benefits of Dilution
Source: ChemSusChem. 2021 Nov 24;15(1):e202102182. doi: 10.1002/cssc.202102182 (PMC9299151; doi:10.1002/cssc.202102182)
Supplement: Supplementary file 1 — Supporting Information [file CSSC-15-0-s001.pdf]

ChemSusChem

Supporting Information

# $LiFePO_4$ Battery Material for the Production of Lithium from Brines: Effect of Brine Composition and Benefits of Dilution

Sara Pérez-Rodríguez, Samuel D. S. Fitch, Philip N. Bartlett, and Nuria Garcia-Araez*© 2021 The Authors. ChemSusChem published by Wiley-VCH GmbH. This is an open access article under the terms of the Creative Commons Attribution License, which permits use, distribution and reproduction in any medium, provided the original work is properly cited.

**Table S1**. Studies on the cycling stability of $LiFePO_4$ electrodes in aqueous electrolytes and brines.

| Electrolyte | $[Li^+]$ / M | Capacity retention / % | Number of charge/discharge cycles | C-rate, specific current or current density | Experimental conditions | Ref. |
|---|---|---|---|---|---|---|
| 1 M $Li_2SO_4$ @ pH = 13 | 2 | 90[a] 85[a] | 1000 50 | 6C, 6 mA $cm^{-2}$ ~C/8, 0.1 mA $cm^{-2}$ | $LiTi_2(PO_4)_3/LiFePO_4$ cell. | [1] |
| 5 M $LiNO_3$ 9 M $LiNO_3$ | 5 9 | 30[a] No relevant capacity fading [a] | 100 | 1C, 170 mA $g^{-1}$ | $LiFePO_4/LiV_3O_8$ cell. | [2] |
| 0.5 M $Li_2SO_4$ | 1 | 62[a] | 10 | 5C, 850 mA $g^{-1}$ | Three-electrode cell using a $LiFePO_4$ electrode and an activated carbon electrode as counter electrode. A saturated calomel electrode was used as reference. | [3] |
| 0.5 M $Li_2SO_4$ Saturated $LiNO_3$ | 0.5 Sat. | 40[b] 80[b] | 500 | 1.1C, 187 mA $g^{-1}$ | $LiFePO_4/Li_{0.47}FePO_4$ cell. | [4] |
| 1.0 M $Li_2SO_4$ | 2 | 82 | 100 | 1C, 170 mA $g^{-1}$ | Three-electrode cell using a $LiFePO_4$ electrode and an activated carbon electrode as counter electrode. An Ag/AgCl electrode was used as reference. Coating the $LiFePO_4$ material with 1 wt. % $AlF_3$ produces a 92% capacity retention. | [5] |
| 1.0 M $Li_2SO_4$ | 2 | 80 | 200 | 10C, 1700 mA $g^{-1}$ | Three-electrode cell using a $LiFePO_4$ electrode and a Pt foil as counter electrode. A saturated calomel electrode was used as reference. | [6] |
| 5 M $LiNO_3$ | 5 | 37 | 6000 | ~12C, 2000 mA $g^{-1}$ | $LiFePO_4$/activated carbon cell. | [7] |
| Saturated $LiNO_3$ | Sat. | 86 | 80 | C/3, 57 mA $g^{-1}$ | $LiFePO_4/VO_2$ cell. | [8] |
| 0.5 M $Li_2SO_4$ | 1 | 73 | 1000 | 2C, 340 mA $g^{-1}$ | $LiMn_2O_4/LiFePO_4$ cell. | [9] |
| 1 M $Li_2SO_4$ | 2 | 91 | 110 | C/10, 17 mA $g^{-1}$ | Three-electrode cell using a $LiFePO_4$ electrode and Pt as counter electrode. An Ag/AgCl electrode was used as reference. | [10] |
| Synthetic Atacama | 0.04 | 21 | 30 | 1C, 170 mA $g^{-1}$ | $LiFePO_4$/NiHCF cell. | [11] |
| Synthetic brine | 0.29 | 84 | 50 | 0.3 mA $cm^{-2}$ | $LiFePO_4/FePO_4$ cell containing an anion exchange membrane. | [12] |

| | | | | | | |
|---|---|---|---|---|---|---|
| 0.5 M $Li_2SO_4$<br>Synthetic Atacama<br>Sunthetic Olaroz<br>Synthetic Altiplano | 1.00<br>0.04<br>0.18<br>0.06 | 86<br>90<br>81<br>79 | 50 | C/10, 17 mA $g^{-1}$ | $LiFePO_4/Li_{0.25}FePO_4$ cell. | **This work** |

[a] Cell assembled in the absence of oxygen in the electrolyte.
[b] After cell assembly, vacuum was applied for ≈1 min to remove trapped air.

**Table S2**. Studies on selective lithium sequestration from brines using $LiFePO_4$ electrodes

| **Brine** | **[$Li^+$] / mM** | **Li purity in brine [a] /%** | **Quantification of lithium sequestration** | | **Experimental conditions** | **Ref.** |
|---|---|---|---|---|---|---|
| | | | **Li uptake in the solid[b] /mg $g^{-1}$** | **Li purity in recovery solution[a]/ %** | | |
| Synthetic sodium rich brine | 50<br>5<br>0.5 | 0.1<br>0.01<br>0.001 | - | 85<br>36<br>21 | 1 cycle of lithium capture (reduction) and release (oxidation) in a $LiFePO_4$/Ag cell, ±0.5 mA $cm^{-2}$, 2 h, recovery solution: 50 mM KCl. | [13] |
| Synthetic Atacama | 42 | 4.1 | - | 99 (-0.05 mA $cm^{-2}$)<br>98 (-0.5 mA $cm^{-2}$)<br>75 (-5 mA $cm^{-2}$) | 1 cycle of lithium capture (reduction, at -0.05, -0.5 or -5 mA $cm^{-2}$) and release (oxidation, +0.5 mA $cm^{-2}$, 60 min.) in a $LiFePO_4$/Ag cell, recovery solution: 50mM $CaCl_2$: . | [14] |
| Natural Atacama | 43 | 4.4 | | 74 | 1 cycle of lithium capture (reduction) and release (oxidation) in a $LiFePO_4$/NiHCF, ±108.5 mA $g^{-1}$, 40 min, recovery solution: 90 mM KCl. | [15] |
| Synthetic West Taijnar salt lake | 32 | 0.7 | 23 | - | 1 cycle of lithium capture (reduction) and release (oxidation), ±1.0 V each step for 10 h, in a $LiFePO_4/FePO_4$ cell containing an anion exchange membrane, supporting electrolyte: 0.5 M NaCl. | [16] |
| Synthetic brine | 29 | 5.4 | 39 | - | 10 cycles of lithium capture (reduction) and release (oxidation), ±0.25 V each step for 10 h, in a $LiFePO_4$/ $FePO_4$ cell containing an anion exchange membrane, supporting electrolyte: 0.5 M NaCl. | [17] |
| Old West Taijnar salt lake brine | 73 | 2.0 | 26 | - | 1 cycle of lithium capture (reduction) and release (oxidation), ±0.2 V each step limited to a current cut-off = 0.05 mA $cm^{-2}$ V, in a $LiFePO_4/FePO_4$ cell containing an anion exchange membrane, supporting electrolyte: 0.5 M NaCl. | [12] |

| | | | | | | |
|---|---|---|---|---|---|---|
| Synthetic Geothermal water | 3.7 | 10.1 | 26<br>(~ 15 mg/g per cycle) | - | 6 cycle of lithium capture (reduction) and release (oxidation), ±0.8 V, each step for 2 h,, in a $LiFePO_4$/KNiFC cell, recovery solution: 200 mg $L^{-1}$ $Na^+$. | [18] |
| 0.5 M $Li_2SO_4$<br>0.025 M $Li_2SO_4$<br>0.0025 M $Li_2SO_4$<br>0.5 M $Li_2SO_4$ +1 M NaCl<br>0.5 M $Li_2SO_4$ +1 M KCl<br>0.5 M $Li_2SO_4$ +1 M $MgCl_2$<br>Synthetic Atacama<br>Synthetic Olaroz<br>Synthetic Altiplano | 1000<br>50<br>5<br>1000<br>1000<br>1000<br>40<br>180<br>60 | 100<br>100<br>100<br>50<br>50<br>50<br>4.0<br>3.3<br>1.3 | 43 (C/10); 37 (1C)[c]<br>43 (C/10); 35 (1C)[c]<br>42 (C/10); 30 (1C)[c]<br>43 (C/10); 35 (1C)[c]<br>43 (C/10); 35 (1C)[c]<br>43 (C/10); 35 (1C)[c]<br>43 (C/10); 26 (1C) [c]<br>43 (C/10); 32 (1C) [c]<br>43 (C/10), 24 (1C) [c] | - | 2 charge/discharge cycles at C/10 (±17 mA $g^{-1}$, 10 h) followed by 5 charge/discharge cycles at 1C (±170 mA $g^{-1}$, 1 h), in a $LiFePO_4$/$Li_{0.25}FePO_4$ cell. | **This work** |

[a] The Li purity equals the concentration of lithium divided by the sum of the concentrations of all other cations. .

[b] The Li uptake equals the mass of lithium absorbed per gram of $FePO_4$ absorbant (mg $g^{-1}$).

[c] Estimated from experimental capacities.

**Table S3**. Molar concentrations of the of the different salts used to modifications of the synthetic Olaroz brine.

| **Brine** | **[LiCl] / M** | **[NaCl] / M** | **[KCl] / M** | **[$MgCl_2$] / M** |
|---|---|---|---|---|
| Synthetic Olaroz | 0.18 | 5.00 | 0.28 | - |
| Synthetic Olaroz containing $MgCl_2$ | 0.18 | 5.00 | 0.28 | 0.11 |
| Synthetic Olaroz containing $MgCl_2$ without NaCl | 0.18 | - | 0.28 | 0.11 |
| Synthetic diluted Olaroz containing $MgCl_2$ | 0.06 | 1.67 | 0.09 | 0.04 |

**Table S4**. Properties of the modifications of the synthetic Olaroz brine.

| Brine | [LiCl] / M | $[Na^+]/[Li^+]$ | $[K^+]/[Li^+]$ | $[Mg^{2+}]/[Li^+]$ | $Li^+$ purity in brine [a] / %[a] | $\eta$ [b] / mPa s | $[Li^+]/\eta$ [c] / M $mPa^{-1}$ $s^{-1}$ | Li uptake [d] / mg $g^{-1}$ | |
|---|---|---|---|---|---|---|---|---|---|
| | | | | | | | | C/10 | 1C |
| Synthetic Olaroz | 0.18 | 27.8 | 1.56 | - | 3.3 | 1.79 | 0.100 | 43 | 32 |
| Synthetic Olaroz containing $MgCl_2$ | 0.18 | 27.8 | 1.56 | 0.6 | 3.2 | 1.83 | 0.098 | 40 | 22 |
| Synthetic Olaroz containing $MgCl_2$ without NaCl | 0.18 | - | 1.56 | 0.6 | 31.6 | 0.90 | 0.201 | 43 | 36 |
| Synthetic diluted Olaroz containing $MgCl_2$ | 0.06 | 27.8 | 1.56 | 0.6 | 3.2 | 1.09 | 0.055 | 42 | 30 |

[a]The Li purity equals the concentration of lithium divided by the sum of the concentrations of all other cations
[b]Dynamic viscosity (mPa s)
[c]Ratio of lithium concentration and dynamic viscosity (M $mPa^{-1}$ $s^{-1}$)
[d]The Li uptake equals the mass of lithium absorbed per gram of $FePO_4$ absorbant (mg $g^{-1}$).

**Comparison of the composition of the synthetic brines used in this work and natural lithium brines**

The experiments here reported were performed in three synthetic brine compositions that represent typical compositions of the lithium reserves in Atacama (Chile),[19,20] Olaroz (Argentina),[21–23] and in Central Altiplano (Bolivia).[24]. The use of synthetic brines facilitates the comparison of results with other research articles, and indeed, tables S1-2 show that most published works used synthetic brines. The synthetic brines used here contained the same concentration of the main cations ($Li^{+}$, $Na^{+}$, $K^{+}$ and $Mg^{2+}$) and anion ($Cl^{-}$) present in the natural brines. The concentration of other minory components ($Ca^{2+}$, $SO_4^{2-}$, $NO_3^{-}$, $BO_3^{3-}$, etc.) depends on the brine location,[24–27] and previous work has shown that the presence of $SO_4^{2-}$ does not compromise the rate or selectivity of the electrochemical sequestration of lithium by $FePO_4$.[28]

**Electrochemical characterization of $LiFePO_4$ electrodes in organic electrolytes**

The electrochemical performance of $LiFePO_4$ electrodes was also studied in organic media using a lithium foil (Rockwood lithium, 100 μm thickness, cut in 12 mm diameter discs) or $Li_{0.25}FePO_4$ electrodes as reference and counter electrodes. $LiFePO_4$ and $Li_{0.25}FePO_4$ electrodes (diameter = 11 mm) in a weight active material:Timcal SUPER C65:PVDF ratio of 8:1:1 were prepared following the same methodology described in the manuscript (see Section 2.2). An aluminium foil (advent research materials, thickness = 0.125 mm, 99%) was used as the substrate for coating the electrodes. The cells were assembled with two glass fibre separators (Whatman, grade GF-F, 12 mm diameter) soaked with 150 μL of 1.0 M lithium hexafluorophosphate solution ($LiPF_6$) in ethylene carbonate (EC) and dimethyl carbonate DMC with volume ratio EC/DMC = 1/1 (LP57, PuriEL) electrolyte. PFA Swagelok type cells were

employed, with aluminium current collectors for the $LiFePO_4$ and $Li_{0.25}FePO_4$ electrodes and copper current collectors for the lithium electrode.

Prior to cell assembly, the electrodes and GF-F separators were dried under vacuum at 120 °C for 48 h in a Büchi tube oven, which was then transferred to an argon filled glovebox without any air exposure. The rest of cell components were dried at 80 ˚C overnight. The cells were assembled in an argon glovebox (≤0.1 ppm $H_2O$; ≤ 0.1 ppm $O_2$). Electrochemical tests were done using galvanostatic charge/discharge cycling of the $LiFePO_4$ cycled against lithium metal or against the $Li_{0.25}FePO_4$ counter electrode, at the specific current of 17 mA h $g^{-1}$ (C/10 rate) using potential limits of 4.1 to 2.7 V vs $Li^+/Li$ and 0.7 to -0.7 V vs. $Li^+/Li_{0.25}FePO_4$, respectively.

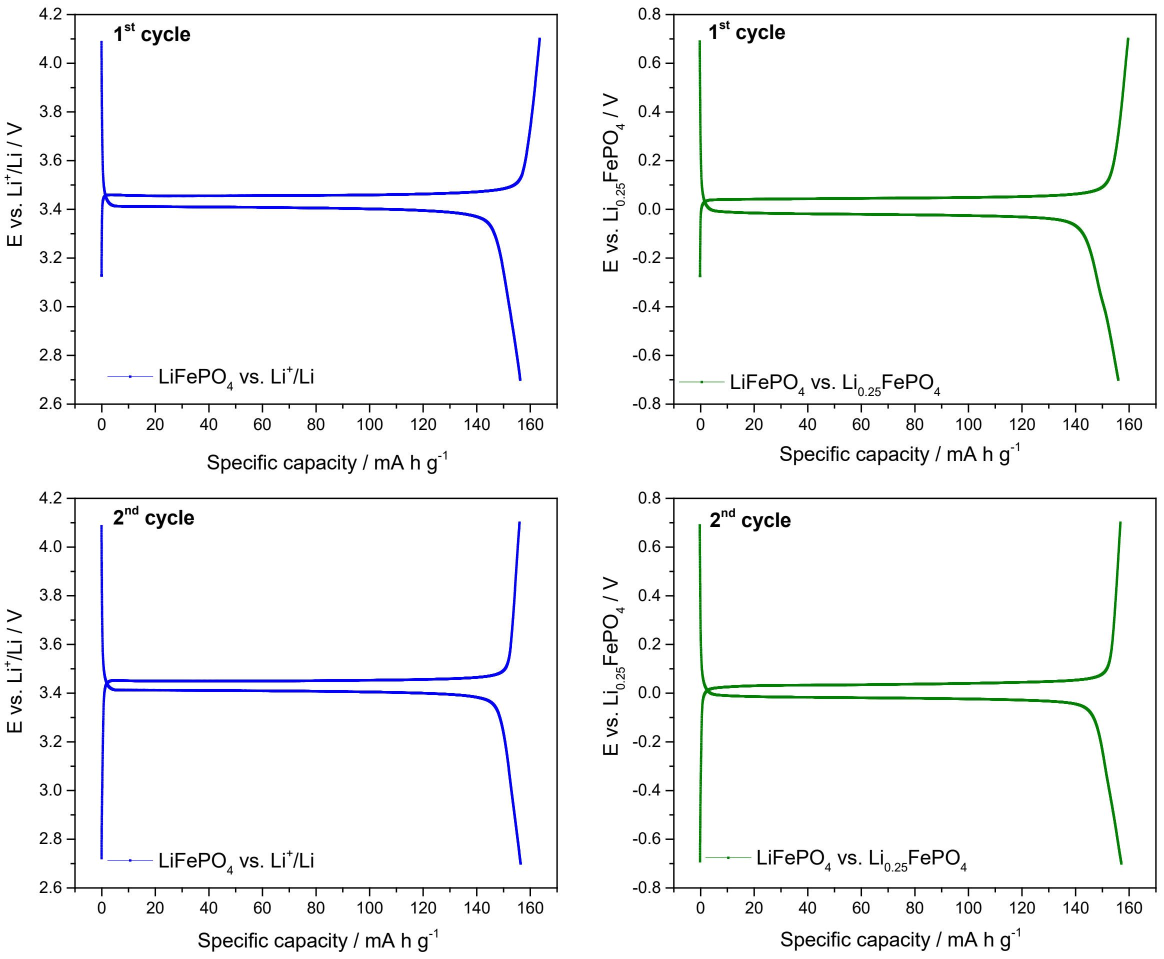


**Figure S1**. First and second cycle charge/discharge profiles of $LiFePO_4$ working electrodes cycled against lithium metal (left: $LiFePO_4$ vs $Li^+/Li$) or against $Li_{0.25}FePO_4$ (right: $LiFePO_4$ vs $Li_{0.25}FePO_4$) counter electrodes. C-rate: C/10. Electrolyte: LP57.

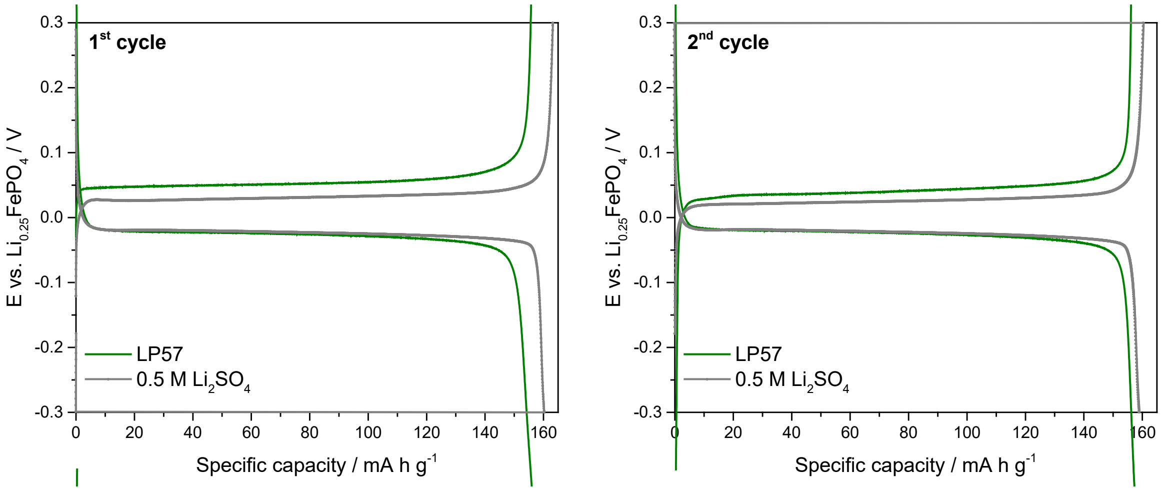


**Figure S2**. First and second cycle charge/discharge profile of $LiFePO_4$ cycled against $Li_{0.25}FePO_4$ at C/10 in LP57 and aqueous 0.5 M $Li_2SO_4$ electrolytes.

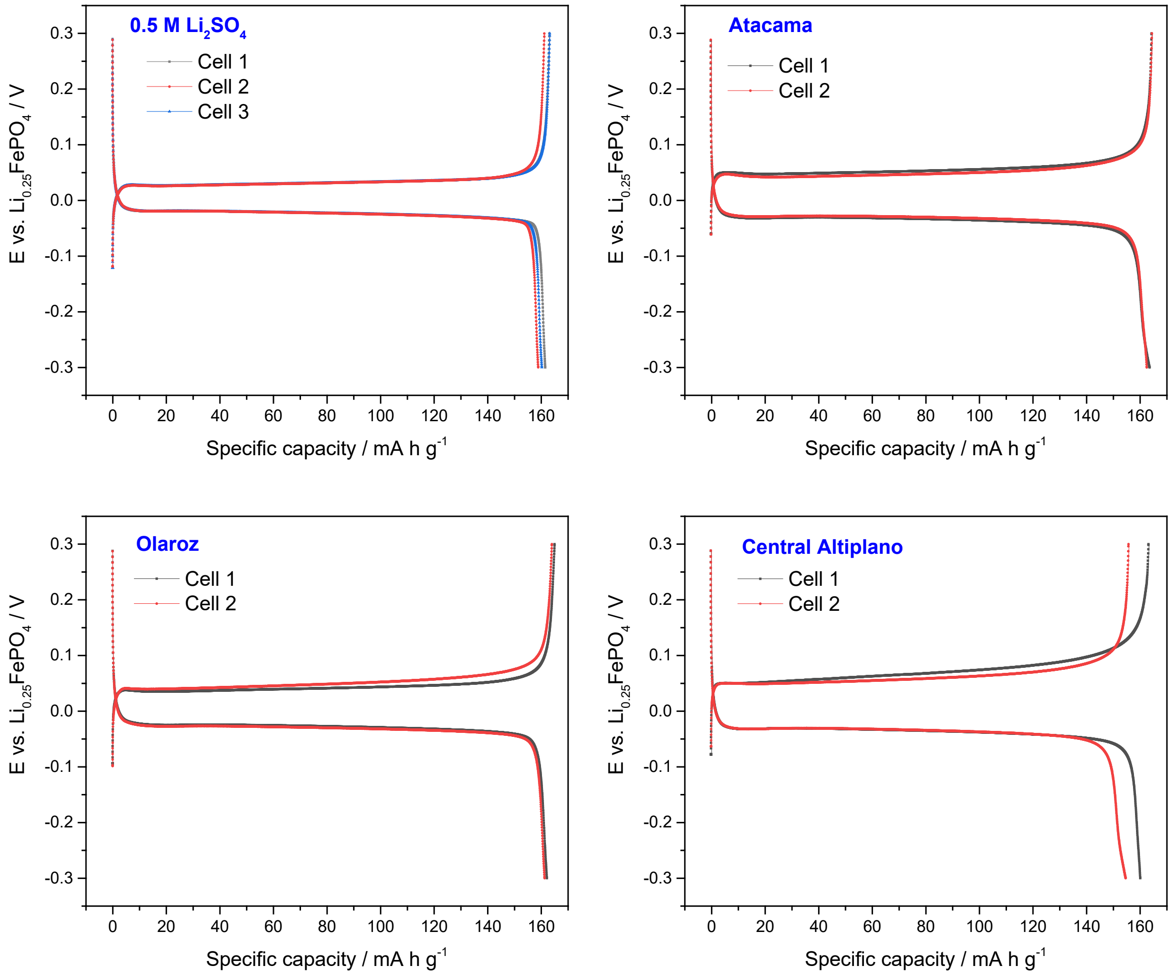


**Figure S3**. Reproducibility of the first charge/discharge cycle recorded at C/10 for $LiFePO_4$ cycled against $Li_{0.25}FePO_4$ in aqueous 0.5 M $Li_2SO_4$ and the different brines.

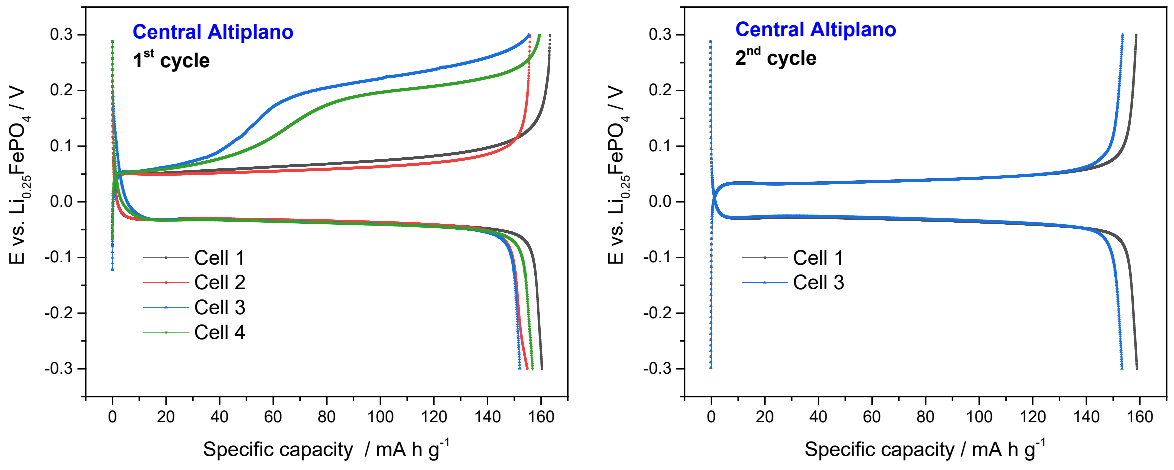


**Figure S4**. Issues with reproducibility of the first charge/discharge cycle with some cells with $LiFePO_4$ cycled against $Li_{0.25}FePO_4$ at C/10 in the Central Altiplano brine. For this brine composition, some of the cells showed an unusual charge voltage profile in the first cycle, which then changed and became the expected charge profile in the second cycle.

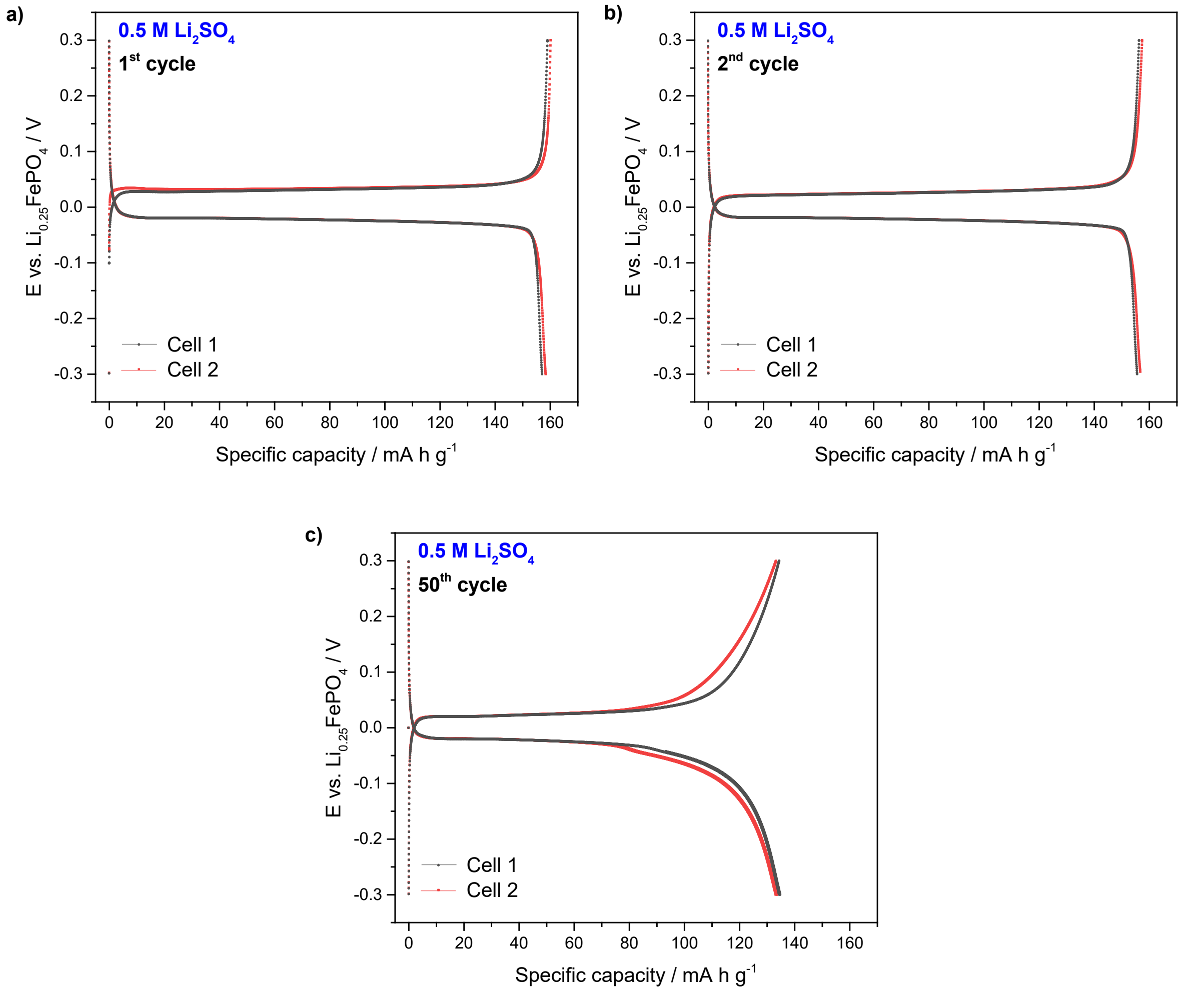


**Figure S5**. Reproducibility of cycling performance of $LiFePO_4$ cycled against $Li_{0.25}FePO_4$ in aqueous 0.5 M $Li_2SO_4$: (a) 1$^{st}$, (b) 2$^{nd}$ and (c) 50$^{th}$ cycles at C/10. The capacity retentions are 86 and 84% for cell 1 and cell 2, respectively.

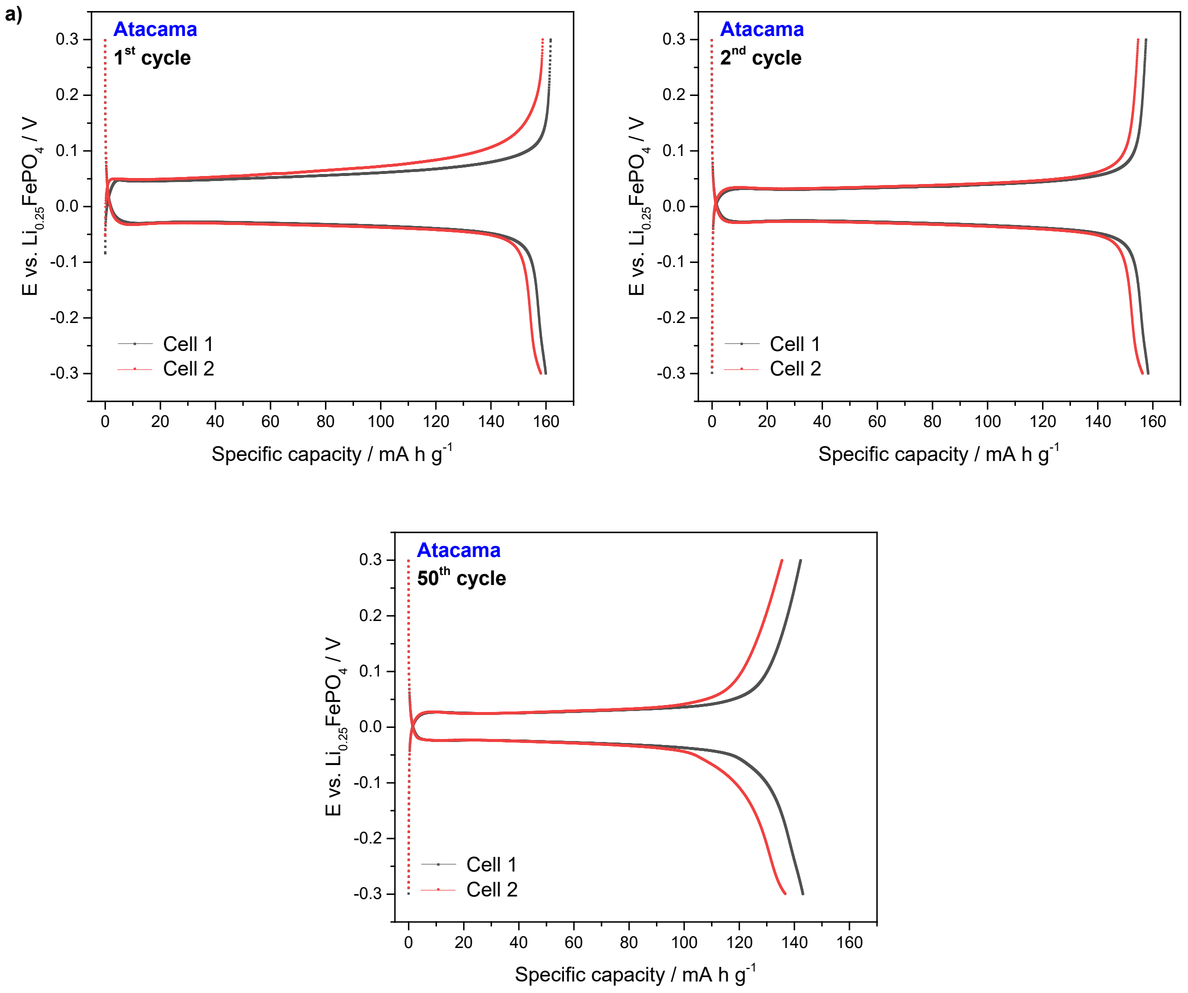


**Figure S6**. Reproducibility of cycling performance of $LiFePO_4$ cycled against $Li_{0.25}FePO_4$ in the Atacama brine: (a) 1$^{st}$, (b) 2$^{nd}$ and (c) 50$^{th}$ cycles at C/10. The capacity retentions are 90 and 87% for cell 1 and cell 2, respectively.

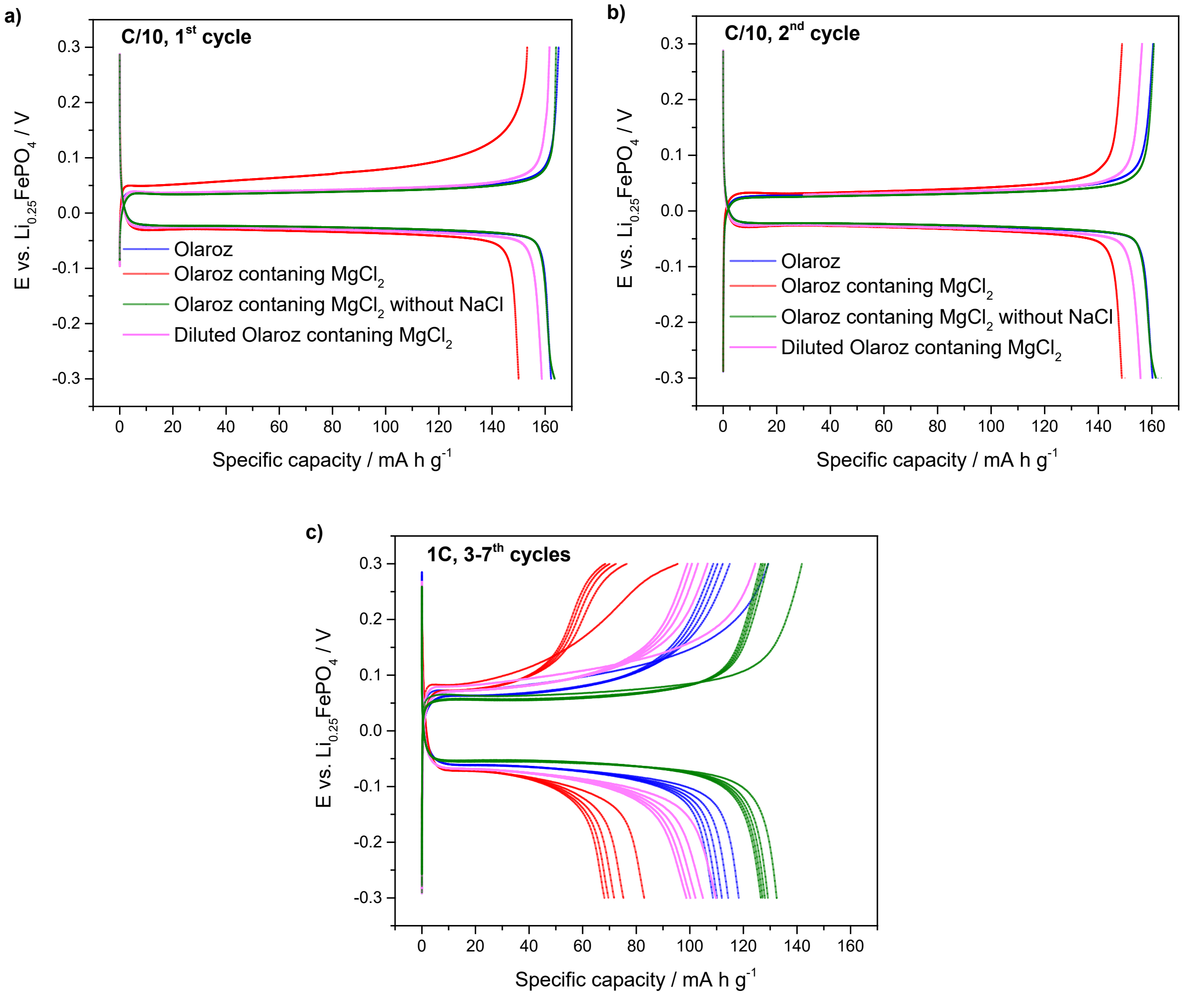


**Figure S7**. (a) 1st and (b) 2nd cycle charge/discharge profile recorded at C/10 and (c) subsequent five cycles at 1C, for $LiFePO_4$ cycled against $Li_{0.25}FePO_4$ in synthetic Olaroz brine (blue curves), synthetic Olaroz brine containing 0.11 M $MgCl_2$ (red curves), and the latter without NaCl (green curves). Magenta curves illustrate the results done in an Olaroz brine containing 0.11 M $MgCl_2$ diluted by three times with water (2 mL of water per mL of the brine). The compositions of the synthetic brines is given in Table S3.

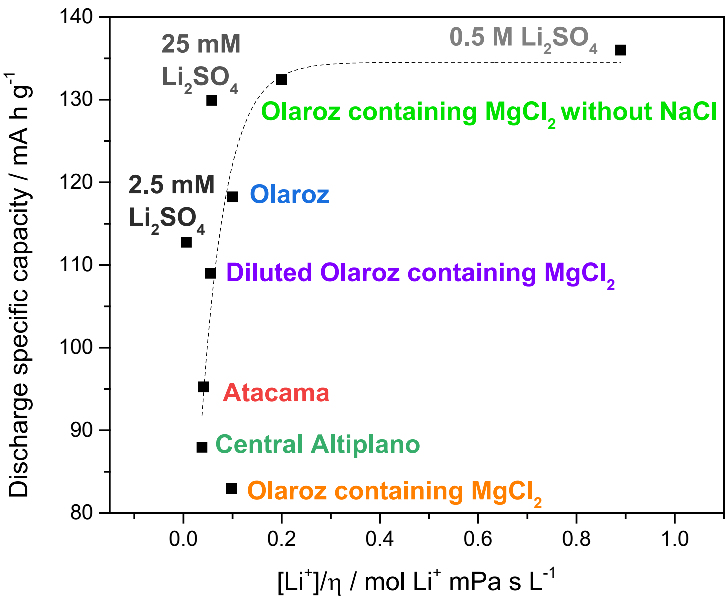


**Figure S8.** Correlation of the first discharge specific capacity at 1C and the ratio of the lithium concentration and viscosity, for the brine compositions reported in the main article (Table 1) and additional brine compositions derived from the Olaroz brine (Table S3).

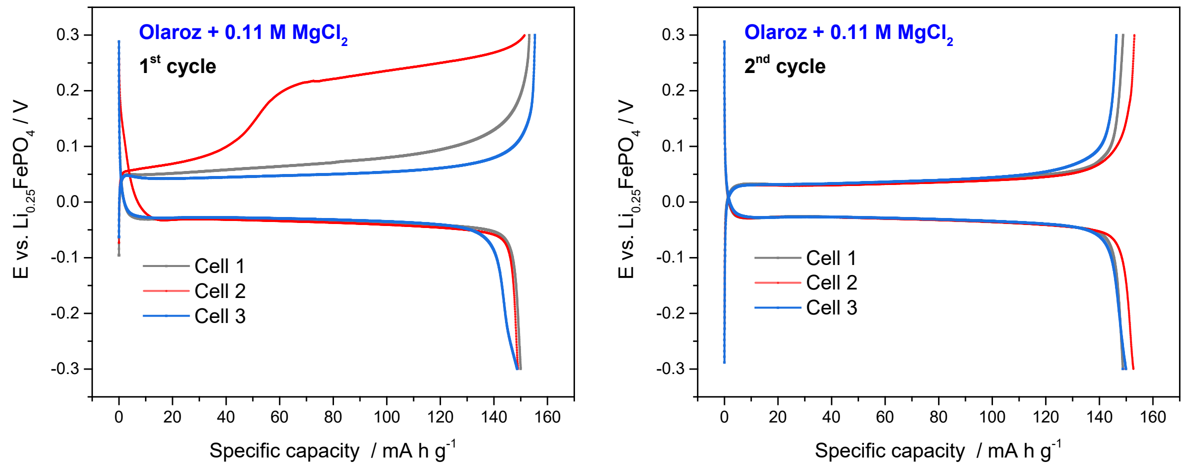


**Figure S9**. Issues with reproducibility of the first charge/discharge cycle with some cells with $LiFePO_4$ cycled against $Li_{0.25}FePO_4$ at C/10 in the Olaroz brine in the presence of 0.11 M $MgCl_2$. For this brine composition, some of the cells showed an unusual charge voltage profile in the first cycle, which then changed and became the expected charge profile in the second cycle. The compositions of the synthetic brines is given in Table S3.

**References:**


[1] J.-Y. Luo, W.-J. Cui, P. He, Y.-Y. Xia, *Nat. Chem.* **2010**, *2*, 760–765.
[2] M. Zhao, B. Zhang, G. Huang, H. Zhang, X. Song, *J. Power Sources* **2013**, *232*, 181–186.
[3] P. He, J.-L. Liu, W.-J. Cui, J.-Y. Luo, Y.-Y. Xia, *Electrochimica Acta* **2011**, *56*, 2351–2357.
[4] D. Gordon, M. Y. Wu, A. Ramanujapuram, J. Benson, J. T. Lee, A. Magasinski, N. Nitta, C. Huang, G. Yushin, *Adv. Energy Mater.* **2016**, *6*, 1501805.
[5] A. Tron, Y. N. Jo, S. H. Oh, Y. D. Park, J. Mun, *ACS Appl. Mater. Interfaces* **2017**, *9*, 12391–12399.
[6] X. Fan, J. Luo, C. Shao, X. Zhou, Z. Niu, *Electrochimica Acta* **2015**, *158*, 342–347.
[7] S. Lv, X. Zhang, P. Zhang, J. Xiang, Y. Li, S. Qiu, C. Qin, *RSC Adv.* **2019**, *9*, 14407–14416.
[8] M. Vujković, I. Stojković, N. Cvjetićanin, S. Mentus, *Electrochimica Acta* **2013**, *92*, 248–256.
[9] X. Zeng, Q. Liu, M. Chen, L. Leng, T. Shu, L. Du, H. Song, S. Liao, *Electrochimica Acta* **2015**, *177*, 277–282.
[10] Y. Zhang, P. Xin, Q. Yao, *J. Alloys Compd.* **2018**, *741*, 404–408.
[11] R. Trócoli, C. Erinmwingbovo, F. La Mantia, *ChemElectroChem* **2017**, *4*, 143–149.
[12] L. He, W. Xu, Y. Song, Y. Luo, X. Liu, Z. Zhao, *Glob. Chall.* **2018**, *2*, 1700079.
[13] M. Pasta, A. Battistel, F. La Mantia, *Energy Environ. Sci.* **2012**, *5*, 9487–9491.
[14] R. Trócoli, A. Battistel, F. L. Mantia, *Chem. - Eur. J.* **2014**, *20*, 9888–9891.
[15] R. Trócoli, A. Battistel, F. La Mantia, *ChemSusChem* **2015**, *8*, 2514–2519.
[16] Z. Zhao, X. Si, X. Liu, L. He, X. Liang, *Hydrometallurgy* **2013**, *133*, 75–83.
[17] X. Liu, X. Chen, Z. Zhao, X. Liang, *Hydrometallurgy* **2014**, *146*, 24–28.
[18] T. Han, X. Yu, Y. Guo, M. Li, J. Duo, T. Deng, *Electrochimica Acta* **2020**, *350*, 136385.
[19] M. S. Palagonia, D. Brogioli, F. La Mantia, *J. Electrochem. Soc.* **2017**, *164*, E586–E595.
[20] M. S. Palagonia, D. Brogioli, F. La Mantia, *Desalination* **2020**, *475*, 114192.
[21] L. L. Missoni, F. Marchini, M. D. Pozo, E. J. Calvo, *J. Electrochem. Soc.* **2016**, *163*, A1898–A1902.
[22] F. Marchini, E. J. Calvo, F. J. Williams, *Electrochimica Acta* **2018**, *269*, 706–713.
[23] F. Marchini, F. J. Williams, E. J. Calvo, *J. Electroanal. Chem.* **2018**, *819*, 428–434.
[24] F. Risacher, B. Fritz, *Chem. Geol.* **1991**, *90*, 211–231.
[25] F. Risacher, H. Alonso, C. Salazar, *Earth-Sci. Rev.* **2003**, *63*, 249–293.
[26] T. Boschetti, G. Cortecci, M. Barbieri, M. Mussi, **2007**, DOI 10.1111/J.1468-8123.2006.00159.X.
[27] R. Lucrecia López Steinmetz, S. Salvi, M. Gabriela García, Y. Peralta Arnold, D. Béziat, G. Franco, O. Constantini, F. E. Córdoba, P. J. Caffe, *J. Geochem. Explor.* **2018**, *190*, 26–38.
[28] N. Intaranont, N. Garcia-Araez, A. L. Hector, J. A. Milton, J. R. Owen, *J. Mater. Chem. A* **2014**, *2*, 6374–6377.
